# Revisiting the model for coactivator recruitment: Med15 can select its target sites independent of promoter-bound transcription factors

**DOI:** 10.1093/nar/gkae718

**Published:** 2024-08-27

**Authors:** Vladimir Mindel, Sagie Brodsky, Hadas Yung, Wajd Manadre, Naama Barkai

**Affiliations:** Department of Molecular Genetics, Weizmann Institute of Science, Rehovot 76100, Israel; Department of Molecular Genetics, Weizmann Institute of Science, Rehovot 76100, Israel; Department of Molecular Genetics, Weizmann Institute of Science, Rehovot 76100, Israel; Department of Molecular Genetics, Weizmann Institute of Science, Rehovot 76100, Israel; Department of Molecular Genetics, Weizmann Institute of Science, Rehovot 76100, Israel

## Abstract

Activation domains (ADs) within transcription factors (TFs) induce gene expression by recruiting coactivators such as the Mediator complex. Coactivators lack DNA binding domains (DBDs) and are assumed to passively follow their recruiting TFs. This is supported by direct AD-coactivator interactions seen *in vitro* but has not yet been tested in living cells. To examine that, we targeted two Med15-recruiting ADs to a range of budding yeast promoters through fusion with different DBDs. The DBD-AD fusions localized to hundreds of genomic sites but recruited Med15 and induced transcription in only a subset of bound promoters, characterized by a fuzzy-nucleosome architecture. Direct DBD-Med15 fusions shifted DBD localization towards fuzzy-nucleosome promoters, including promoters devoid of the endogenous Mediator. We propose that Med15, and perhaps other coactivators, possess inherent promoter preference and thus actively contribute to the selection of TF-induced genes.

## Graphical Abstract

**Graphical Abstract:**
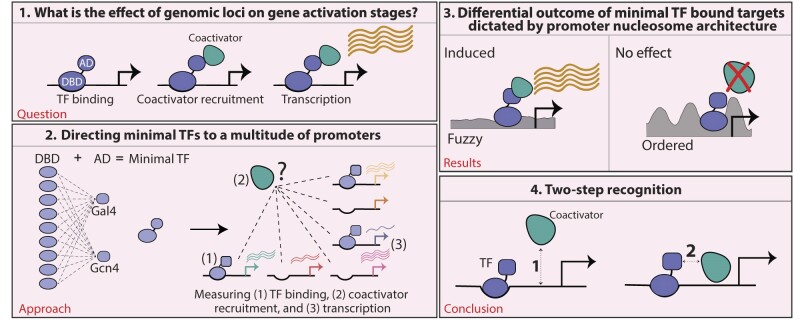

## Introduction

Transcription factors (TFs) contain DNA binding domains (DBDs) that recognize specific sequence motifs and activation domains (ADs) that recruit coactivators to TF-bound locations (1–6). In eukaryotes, DBDs and ADs occupy small portions of the TF sequence, with typical domain sizes of 30–60 AAs within hundreds or even thousands of TF residues. Still, classical experiments have shown that direct fusions of DBDs to ADs generate minimal TFs capable of activating reporter gene expression (1,7), consistent with their ability to interact with DNA and coactivators, respectively, outside cells (8–10). Over the past decades, DBD-AD fusions have been used to define DBD motif preferences, screen for protein-protein interactions, and reprogram gene expression (11–13).

Minimal TFs were employed extensively to study the properties of ADs and coactivators inside cells by monitoring the transcriptional outcomes of thousands of DBD-fused elements using individual reporter genes (14–21). The binding of these fused factors to the tested regulatory regions was not measured but was assumed to strictly follow the recruiting DBD (1–4,14–16,18–28). This assumption follows the prevailing model of coactivator recruitment but has not yet been tested within the genomic context.

Several results have led us to revisit the assumption that ADs and coactivators lack inherent promoter preferences. First, our recent mapping of binding determinants across TF sequences pointed to weak effects originating from sequences overlapping predicted ADs (29–32). Second, plasmid-based screens revealed that the activity of DBD-recruited TFs (26) or cofactors (27) differs across regulatory regions. While this was attributed to downstream interactions (5,6,26,27,32,33), we reasoned that it could also result from differential DNA binding. Finally, several recent reports suggested reciprocal interactions between TFs and cofactors (34–36).

Here, we systematically examined the interplays between DBDs, ADs and the Med15 coactivator in defining binding localization and transcription activities across the genome. We extended previous analyses of minimal DBD-AD TFs in four principal ways. First, we controlled for TF abundances using libraries of synthetic promoters. Second, we characterized transcriptional changes at the genomic scale. Third, we mapped the DNA binding locations of all tested factors and the recruited Med15 coactivator. Finally, we analyzed the direct outcomes of DBD-Med15 fusions on genomic binding and gene induction. We find that only a subset of promoters bound by the minimal DBD-AD TFs, characterized by a fuzzy nucleosome architecture, permit Med15 binding and are transcriptionally induced. Furthermore, rather than being passively recruited, we provide evidence that Med15 actively contributes to the selection of induced genes through inherent preferences of localizing to a subset of fuzzy nucleosome promoters.

## Materials and methods

### Experimental design of strains

All TF constructs generated in this study were inserted into the HO locus using CRISPR; an exception is the expression library for Msn2 and its derivative TFs that were inserted into the Msn2 native locus and the DBD_Msn2/Gcn4_-Med15 fusions presented in Figure 7B-D that were constructed in the Med15 locus.

All strains used in this study were built based on this sequence: TF-MNase-SV40NLS-Adh1 terminator (Supplementary Figure S1C). In strains used for fluorescent experiments, a YFP was lifted from pBS7 (Addgene plasmid #83 774) and fused to the TF downstream of the NLS. Supplementary Table S1 contains the information on all strains used in this study, the assays performed, and the figures in which relevant data is shown.

### Data analysis

Data analysis was performed using Python (version 3.83) using Jupyter Notebooks (37). The Pandas (38), NumPy (39, Matplotlib (40) and Seaborn (41) libraries were utilized for data manipulation, numerical computations, and visualization. Statistical analyses were conducted employing the SciPy library (42). Data related to biological sequences was analyzed with BioPython (43). Pre-processing of NGS-related data was done using Snakemake (44). Additionally, custom scripts were developed to preprocess, clean and analyze the collected datasets.

### Budding yeast growth, maintenance and genetic manipulation

All genetic manipulations were performed on the *S. cerevisiae* BY4741 strain (genotype: MATa his3Δ1 leu2Δ0 met15Δ0 ura3Δ0 genotype) (45) using CRISPR. Transformations were performed using the LiAc/SS DNA/PEG method (46). Following validation by Sanger sequencing, the pbRA89 (addgene plasmid #100 950) carrying the CRISPR-Cas9 system from positive colonies were lost by growth in YPD (yeast extract peptone dextrose) and selection for colonies without bRA89-encoded Hygromycin resistance. Ligation of the gene-specific guide-RNA into the bRA89 plasmid was performed as previously described (47). Gene deletion strains used in this study were generated by inserting the amplification product of a NatMX6 cassette, derived from the pAG25 (addgene plasmid #35 121), or a KanMX cassette from the pBS7 instead of the ORF of the deleted protein. All strains generated for this study were verified using PCR and gel electrophoresis followed by Sanger DNA sequencing. The primers used for generating the strains used in this study are found in Supplementary Table S1.

### Cell growth before experiments

Yeast strains were freshly thawed from frozen stock, plated on YPD plates and grown. Single colonies were picked and grown at 30°C in liquid SD (synthetic complete with dextrose) medium overnight, reaching stationary phase OD_600_ ≈10, then diluted again into fresh SD medium for the experiment.

### ChEC-seq experiments

The experiments were performed as described previously (48), with some modifications. The stationary cultures described above were diluted ∼2 × 10^3^-fold into 12 ml fresh SD media and grown overnight to reach an OD_600_ of 0.4 the following. Cultures were pelleted at 1500g for 2 min and resuspended in 0.5 ml buffer A (15 mM Tris pH 7.5, 80 mM KCl, 0.1 mM EGTA, 0.2 mM spermine, 0.5 mM spermidine, 1× cOmplete EDTA-free protease inhibitors [Roche, one tablet per 50 ml buffer], 1 mM PMSF) and then transferred to 2 ml 96-well plates (Thermo Scientific). Cells were washed twice in 1 ml Buffer A. Next, the cells were resuspended in 150 μl Buffer A containing 0.1% digitonin, transferred to an Eppendorf 96-well plate (Eppendorf), and incubated at 30°C for 5 min for permeabilization. Next, we added CaCl_2_ to a final concentration of 2 mM to activate the MNase and incubated it for 30 s exactly. The MNase treatment was stopped by adding an equal volume of stop buffer (400 mM NaCl, 20 mM EDTA, 4 mM EGTA and 1% SDS) to the cell suspension. After this, the cells were treated with Proteinase K (0.5 mg/ml) at 55°C for 30 min. An equal volume of phenol–chloroform pH 8 (Sigma-Aldrich) was added, vigorously vortexed, and centrifuged at 17 000*g* for 10 min to extract DNA. Following the extraction, the DNA was precipitated with 2.5 volumes of cold 96% EtOH, 45 mg Glycoblue, and 20 mM sodium acetate at –80°C for >1 h. Next, the tubes were centrifuged (17 000*g*, 4°C for 10 min), the supernatant was removed, and the DNA pellet was washed with 70% EtOH. The DNA pellets were dried and resuspended in 30 μl RNase A solution (0.33 mg/ml RNase A in Tris-EDTA [TE] buffer [10 mM Tris and 1 mM EDTA]) and treated at 37°C for 20 min. DNA cleanup was performed using SPRI beads (Ampure XP, Beckman Coulter) to enrich for small DNA fragments and remove large DNA fragments that might result from spontaneous DNA breaks. First, a reverse SPRI cleanup was performed by adding 0.8× (24 μl) SPRI beads, followed by 5 min incubation at RT. The supernatant was collected, and the remaining small DNA fragments were purified by adding additional 1× (30 μl) SPRI beads and 5.4× (162 μl) isopropanol and incubating 5 min at RT. The beads were washed twice with 85% EtOH, and small fragments were eluted in 30 μl of 0.1× TE buffer.

### Chec-Seq next-generation sequencing library preparation

Library preparation was performed as described in (49) with modifications. Following RNase treatment and reverse SPRI cleanup, the DNA fragments served as an input to an end-repair and A-tailing (ERA) reaction. 5.4 μl ERA reaction was prepared (1 × T4 DNA ligase buffer [NEB], 0.5 mM dNTPs, 0.25 mM ATP, 2.75% PEG 4000, 6U T4 PNK [NEB], 0.5U T4 DNA Polymerase [Thermo Scientific], 0.5U Taq DNA polymerase [Bioline]) and added to 14.6 μl of each sample and incubated for 20 min at 12°C, 15 min at 37°C and 45 min at 58°C in a thermocycler. After the ERA reaction, reverse SPRI cleanup was performed by adding 0.5× (10 μl) SPRI beads (Ampure XP, Beckman Coulter). The supernatant was collected, and the remaining small DNA fragments were purified with additional 1.3× (26 μl) SPRI beads and 5.4× (108 μl) isopropanol. After washing with 85% EtOH, small fragments were eluted in 17 μl of 0.1 × TE buffer; 16.4 μl elution was taken into 40 μl ligation reaction (1 × Quick ligase buffer [NEB], 4000U Quick ligase [NEB], and 6.4 nM Y-shaped barcode adaptors with T-overhang (50) and incubated for 15 min at 20°C in a thermocycler. After incubation, the ligation reaction was cleaned by performing a double SPRI cleanup: first, a regular 1.2× (48 μl) SPRI cleanup was performed and eluted in 30 μl 0.1 × TE buffer. Then instead of separating the beads, an additional SPRI cleanup was performed by adding 1.3× (39 μl) HXN buffer (2.5 M NaCl, 20% PEG 8000) and final elution in 24 μl 0.1 × TE buffer; 23 μl elution were taken into 50 μl enrichment PCR reaction (1 × Kappa HIFI [Roche], 0.32 μM barcoded Fwd primer and 0.32 μM barcoded Rev primer, (50) and incubated for 45 s in 98°C, 16 cycles of 15 s in 98°C and 15 s in 60°C, and a final elongation step of 1 min at 72°C in a thermocycler.

The final libraries were cleaned using 1.1× (55 μl) SPRI and eluted in 15 μl 0.1 × TE buffer. Library concentration and size distribution were quantified by Qbit (Thermo Scientific) and TapeStation (Agilent), respectively. For multiplexed next-generation sequencing (NGS), all barcoded libraries were pooled in equal amounts, and the final pool was diluted to 2 nM and sequenced on NovaSeq 6000 (Illumina). Sequence parameters were Read1: 61 nucleotides (nt), Index1: 8 nt, Index2: 8 nt, Read2: 61 nt.

### TF-promoter library preparation

The libraries were constructed using strains in which the TF of interest is overexpressed from the HO locus and fused to a YFP. In all libraries, the respective native TF was deleted from the genome. A set of 192 synthetic promoters from (22) was used to generate the libraries. The promoters were pooled and transformed to replace the TEF1 promoter, overexpressing the TF. For the Msn2-related libraries, the same was done in the native locus, and the promoters from the library replaced the native promoter. The synthetic promoters were lifted together with the URA3 marker, and selection was made on plates lacking Uracil. For each library, we collected approximately 200 colonies after the transformation. Each of them was reisolated two times to ensure that each colony originated by a single transformant and measured YFP fluorescence using a flow cytometer (BD LSRII system from BD Biosciences, San Jose, CA, USA) in two repeats with excitation at 488 nm and emission at 525 ± 25 nm. We picked up to 50 strains for each library that spanned the expression range, had unimodal fluorescence distributions, and were highly similar between the two repeats.

### Chec-seq NGS data processing

Raw reads from ChEC-seq libraries were demultiplexed using bcl2fastq (Illumina), and adaptor dimers and short reads were filtered out using cutadapt with parameters: ‘−O 10 –pair-filter = any –max-n 0.8 –action = mask’. Filtered reads were subsequently aligned to the *S. cerevisiae* genome R64-1-1 using Bowtie 2 (51) with the options ‘‐‐end-to-end ‐‐trim-to 40 ‐‐very-sensitive’. The genome coverage of fully aligned read pairs was calculated with GenomeCoverage from BEDTools (52) using the parameters ‘-d –5 –fs 1’, resulting in the position of the fragment ends, which correspond to the actual MNase cutting sites. The reads were normalized to 107.

### Promoter definition

Promoters were defined only for genes with an annotated transcript, as described before (29). The length of each promoter was defined as 700 bp upstream to the transcription start site (TSS) or to the position where a promoter meets another transcript. The signal across each promoter was summed and normalized to the maximal promoter length (700 bp), $\frac{{sum( {signal\ on\ promoter} )}}{{len( {promoter} )}}*700$ to calculate the overall promoter binding for each sample.

### Target promoter definition

**Figure 1.**
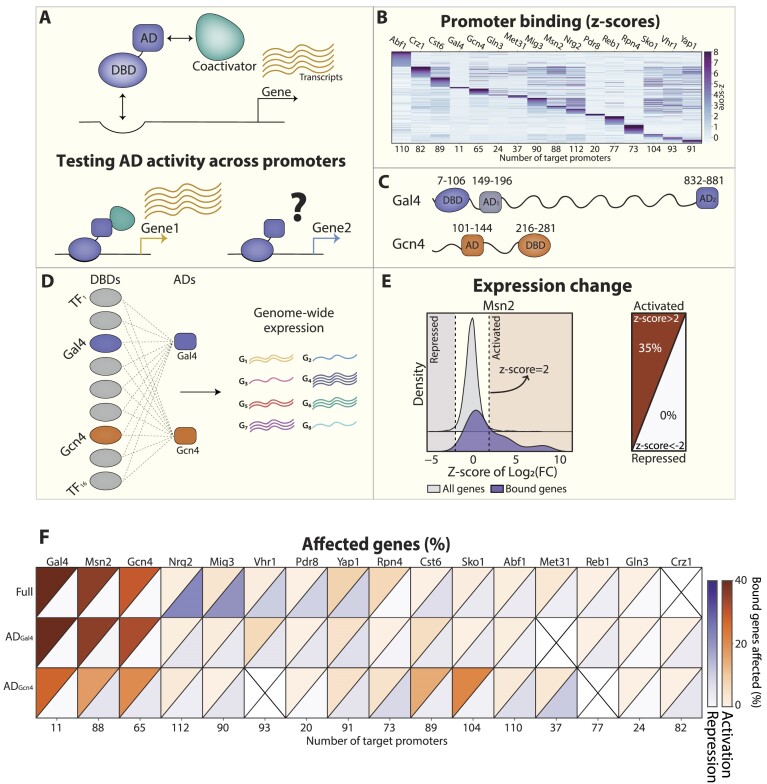
Targeting ADs to hundreds of promoters through DBD fusions. (A–D) Minimal TFs composed of a DBD-AD fusion can be used to compare the function of activation domains (ADs) at different promoters: TFs induce gene expression by recruiting general coactivators such as Med15 (**A**, top). We asked whether this capacity varies between promoters (A, bottom). To direct ADs to different genomic loci, we used our lab data to select 16 DBDs corresponding to TFs occupying a wide range of promoters. Shown in (**B**) is the *z*-score transformed binding signal of the selected TFs (columns) at individual promoters (rows, methods). This analysis includes all 672 promoters bound by at least one selected factor (methods). We then chose two well-characterized ADs belonging to Gal4 and Gcn4 (**C**, AD_2_ of Gal4 was used). The 32 minimal TFs corresponding to all DBD-ADs (**D**, left) were then engineered and integrated, together with the 16 full TFs, into the genome downstream of a strong TEF1 promoter while fused to a nuclear localization signal (NLS, Supplementary Figure S1C). Of the 48 desired TFs, 44 were successfully generated and analyzed through genome-wide expression profiling (D, right, Supplementary Table S1). (E, F) Fraction of wild-type TF-bound genes affected by each tested factor: The set of TF-bound targets was defined based on the binding profiles of the endogenously expressed full TFs (methods). Transcriptional changes were defined by comparing gene expression during exponential growth in the respective TF over-expressing strain to that of wild-type cells. Shown in (**E**, left) is the distribution of expression changes of all TF-bound genes (purple) compared to all other genes (grey), exemplified by Msn2. The fraction of induced (or repressed) genes was then defined as those showing significant change as compared to the background distribution (*z*-score > 2, methods), as summarized by the triangles on the right. Shown in (**F**) are the fractions of induced (upper triangle, brown) and repressed genes (lower triangle, blue) for all tested factors. A black ‘X’ marks missing strains.

To define the target promoters for a given TF, we first calculated the sum of signal on each promoter as defined above (see Promoter definition). *Z*-scores were then calculated for the sum of signals over all promoters. The promoters with z-score >= 3 were defined as targets for the respective TF. Promoters in (Figures 1B, 3B, and Supplementary Figure S10A) were ordered according to the binding strength of the first presented TF up to the point where the value goes below 3 (in units of Z-score). After that, the ordering was done according to the binding strength of the next TF (until it goes below 3). In Figures 5C, 6C, F, Supplementary Figure S13A, and S15C, promoters were ordered according to the descending OPN score of each set of TF-bound promoters.

### Calibration of absolute TF binding strength

For measuring absolute TF binding strengths presented in Supplementary Figure S6, a spike-in of a yeast strain containing a fusion between the Aro80 TF and MNase was added to a sample during the Chec-Seq experiment to calibrate the binding strength of transcription factors. The Aro80 TF was selected for calibration because of its unique restrictive binding pattern demonstrating signal localization to only three bidirectional gene promoters (Aro9, Aro10, Naf1) (53), enabling the separation of its signal from any other tested TF. The Aro80-MNase strain was grown in liquid SD media in a large flask parallel to the experimental samples described above. Following OD measurements, liquid media containing the Aro80-MNase strain was added to the sample in such a way that it would represent 10% of the yeast population in each sample tube. Following this step, the ChEC-Seq experimental protocol proceeded with no change.

### Binding strength calculations with Aro80-MNase spike-in

Samples containing Aro80-MNase spike-in were preprocessed as above (described in the ChEC-Seq processing and analysis method). After sample normalization and calculations of the sum signal on promoters, the ratio between the sum over all other promoters to the Aro80 TF target promoters (ARO9, ARO10, NAF1) in a given sample was calculated. This ratio represents the total binding strength of a given TF.

### RNA sample collection and extraction

Six dilutions of cells were grown overnight in liquid SD media in 96 Deep Well plates and then diluted using a Tecan Robot. Wells with OD_600_ closest to 0.4 were collected, transferred to a new 96-plate, and centrifuged for 60 s at 4000*g*. The supernatant was removed, and pellets were frozen in liquid nitrogen and stored at −80°C until RNA preparation. If fluorescence measurements were performed, 120 ul of samples were aliquoted into a new 96U plate and immediately transferred to a BD LSR II FACS machine, and the process proceeded as detailed below. mRNA was extracted using a modified protocol of the nucleospin 96 RNA kit (Macherey-Nagel, Duren, Germany). Specifically, cell lysis was done in a 96-deep-well plate by adding 450 μl of lysis buffer containing 1 M sorbitol (Sigma-Aldrich), 100 mM EDTA, and 0.45 μl lyticase (10 IU/μl). The plate was incubated at 30°C for 30 min to break the cell wall and then centrifuged for 10 min at 2500 rpm, and the supernatant was removed. From this point, extraction proceeded as in the nucleospin 96 RNA kit protocol, only substituting β-mercaptoethanol with DTT 1 M.

### RNA library preparation for next-generation sequencing

RNA libraries were created as described before (54): poly(A) RNA was selected by reverse transcription with a barcoded poly(T) primer. The barcoded DNA–RNA hybrids were pooled and fragmented by a hyperactive variant of the Tn5 transposase. Tn5 was stripped off the DNA by treatment with SDS 0.2%, followed by SPRI beads clean up, and the cDNA was amplified and sequenced with the Illumina NovaSeq 6000 (same parameters as above).

### Processing and analysis of RNA-seq data

We mapped 50-bp reads of the RNA-seq of every sample to the *S*. *cerevisiae* genome (R64-1-1) using bowtie2 (parameters:-p8 –local –very-sensitive –trim-to 30). After alignment to the genome, samples with <200 000 reads were discarded from the analysis to prevent an artificial enrichment for highly expressed genes. For every sequence, we normalized for PCR bias using the unique molecular identifier (UMI), scoring each position on the genome by the unique number of UMIs it had out of all possible UMIs. To quantify the expression of each gene, we summed all reads aligning from 400 bp upstream to the stop codon and 200 bp downstream of it to include 3′ UTRs. The number of reads for each sample was normalized to 1e6.

### Flow cytometry measurements of TF promoter libraries

For this experiment, we used TF-YFP-tagged strains (see TF-Promoter library preparation). Cells were grown for the RNA-seq experiment (see RNA sample collection and extraction), and 120 ul of samples were collected into a new 96U plate as described above. After that, fluorescence was measured using a flow cytometer. Flow cytometry measurements and analysis were done using the BD LSRII system (BD Biosciences). Flow cytometry was conducted with excitation at 488 nm and emission at 525 ± 25 nm. The average number of cells analyzed was ∼25 000. We filtered outliers by clustering all cells from experiments using the cytoflow software (55) with a Gaussian mixture into two populations, assuming that clusters would represent cells and noise. We took cells that fell into two standard deviations from the mean of the defined cell mixture. To eliminate the background fluorescence, we subtracted the log-transformed median fluorescence of at least four repeats of a non-fluorescent BY4741 from the log-transformed fluorescence distributions.

### Preliminary gene expression filter

To ensure the robustness of our analysis and improve the reproducibility of our results, we filtered our expression data to exclude low-expressing and noisy genes from our dataset. Only genes with at least 4.5 log_2_ normalized reads in at least 2.5% of our samples (27/1076) were retained (5013 out of 6649 genes).

### Library gene correlations analysis

After normalizing gene counts and FACS values in any given coupled RNA-seq/FACS experiment, vectors representing the log_2_ normalized mRNA counts of each strain were ordered by the respective fluorescence values in the respective TF-expressing strain. For each gene within each library, we calculated the Spearman rank correlation value and the corresponding p-value between its expression level and the abundance of the relevant TF. *P*-values were filtered using the Benjamini–Hochberg (FDR) correction to account for multiple comparisons.

### Differential gene expression of TEF1 overexpression strains

To calculate the fraction of induced/repressed genes shown in Figure 1E and Supplementary Figure S10E, we needed to detect differentially expressed genes out of the set from the bound ones. For that, we first defined the expression level of each gene in exponentially growing cells using >60 BY4741 mRNA samples collected for this study. Following the read normalization and log2 transformation, the median of each gene over all samples was used to represent its steady-state expression level. Then, this vector was subtracted from the log_2_ transformed normalized counts of each TEF1-(minimal/Full TF) sample to get the log_2_ fold-change of each gene upon TF overexpression. The genes were divided into 10 equally sized bins based on their steady-state expression level calculated above, and the mean and standard deviations were calculated inside each such bin in each sample. To detect the differentially expressed genes within the subset of bound genes in each sample, we conducted a t-test within each bin for the bound versus unbound gene sets. Genes with *z*-score >2 were defined as differentially expressed.

### Library means scatterplots and gene induction heatmaps normalization

**Figure 2.**
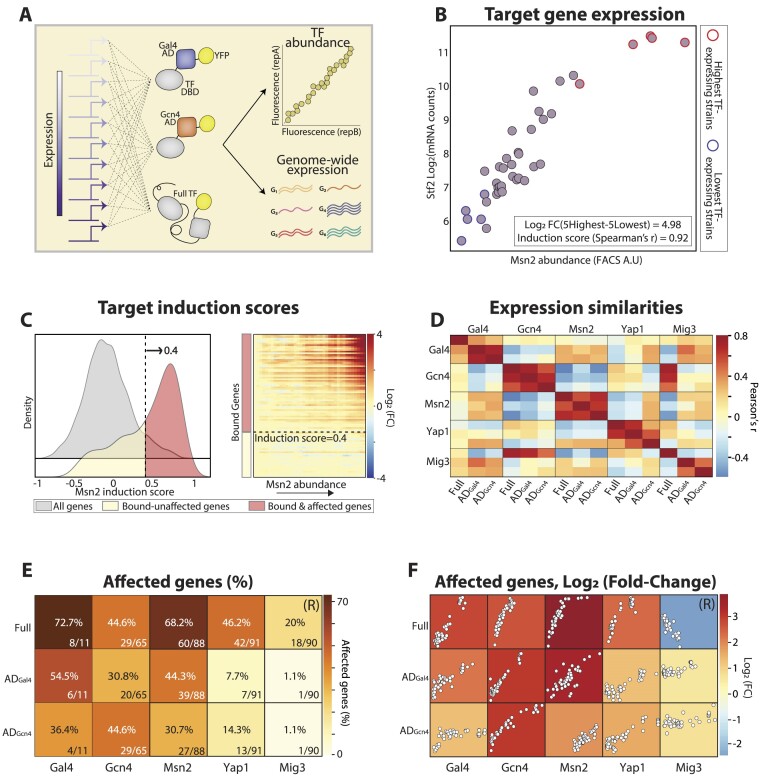
TF promoter libraries allow sensitive quantification of target gene expression. (**A**) Controlling TF abundance using a library of synthetic promoters: to enable a quantitative analysis of induced genes, we used synthetic promoters to engineer libraries of strains expressing the TF of interest at gradually increasing abundances, as quantified through YFP fusion (methods). For each of the five selected factors, we generated three libraries expressing the two DBD-AD fusions and the full TF at increasing levels and analyzed all strains through genome-wide expression profiling. (B, C) Gene induction scores quantify the expression response driven by increasing TF abundance: expression levels of TF-target genes increase gradually with TF abundance, as shown for the Msn2 library, focusing on STF2, an Msn2-bound gene (**B**) and all Msn2-bound targets (**C**). Each dot in (B) represents a strain positioned according to its measured Msn2-YFP abundance (x-axis) and STF2 expression levels (y-axis). The log2 fold-change between the 5 lowest and highest Msn2 expressing strains is shown at the bottom right (4.98) together with the calculated correlation (Spearman's *r* = 0.92). This correlation between the expression levels of a target gene and the abundance of the TF was defined as the induction score. We used the FDR-adjusted p-value of the correlation as our threshold for significant induction (Spearman's *r* ≈ 0.4, C, left, methods). All Msn2-bound targets are shown in (C, right), ordered by their induction scores (rows), while strains are ordered by Msn2 abundance (columns). See Supplementary Figure S2B for all other full and minimal TFs. (**D**) Correlation of gene induction between full and minimal TFs: the similarity (Pearson's *r*) in gene induction scores across all 15 libraries is shown, as indicated. This analysis includes all genes receiving a significant induction score in at least one library of a full TF from the set of genes bound by the native TFs (methods). Note the expected negative correlation between the repressor Mig3 and the DBD_Mig3_-AD fusions. (E, F) The activity of full and minimal TFs at promoters bound by native TFs: shown are the fraction of TF-bound genes receiving a significant induction/repression score (**E**) and the average log2 fold-change of those genes (**F**, color). The dots in F show the average expression of TF-bound genes within individual library strains as a function of TF abundance. For all TFs, except for the full Mig3, the analysis was performed on induced genes. As Mig3 is a repressor, we focused on its down-regulated targets, as indicated by (R).

For the heatmaps found in Figure 2C and Supplementary Figure S2B, each library was normalized internally by its mean expression signal for each gene. Then, the mean of the five strains with the lowest abundance was subtracted from the result to reposition low-abundant strains closer to 0 for easier library comparison. The genes were ordered for each set of same-DBD TFs based on the fold-change in the respective full TF library (defined by the delta between the mean of the five highest and five lowest TF-expressing strains). The data was smoothed by averaging values in a rolling window of three strains. For the scatterplots in Figure 2F, the data was normalized as described above, and the expression changes of the affected genes out of the respective native TF-bound targets were averaged inside each library.

### Correlation of gene expression changes between libraries

The analysis in Figure 2D was performed on the union of all native TF targets that passed the induction filter in each of the full TF libraries – Msn2, Gal4, Gcn4, Yap1 and Mig3 (see Library gene correlations analysis). To analyze and compare the different expression profiles, we calculated the Spearman rank correlation of each gene from the given set to the abundance of the TF in each of the 15 libraries, converting the complex gene expression profiles into one number for each gene. Next, Pearson's *r* correlation between the obtained Spearman correlation vectors was calculated and shown as a heatmap.

### Motif score analysis, probability weight matrices (PWM) and seqlogos from ChEC-seq data

All possible x-mer sequences were given a numerical index for the motif analysis in total $\frac{{{{4}^x}}}{2}$ (forward and reverse complement forms of each x-mer were given the same index). Each nucleotide in the yeast genome was indexed according to the x-mer that begins from it, and the signal around mid-position was averaged (20 bp window) to score each x-mer occurrence. The averaged signal for each x-mer was then calculated across all its occurrences in all promoters and assigned as its relative binding score.

As calculated above, PWMs of the different TFs were generated based on the ten most bound 7-mers of each factor. The sequences were then aligned to the top bound motif using the Needleman-Wunsch local alignment algorithm. Each motif contributed to the PWM based on its relative binding score. Sequence logos were plotted using the logomaker (56) package.

### Obtaining signals of *in-vitro* motifs

For the analysis of motif binding, PWMs were taken from the CIS-BP database (57) (http://cisbp.ccbr.utoronto.ca/), and their occurrences in the genome were detected using the FIMO software (58) from the MEME suite, normalizing for background counts of nucleotide in the S. Cerevisiae genome. Due to a lack of data regarding the Vhr1 motif, the motif of its paralog, Vhr2, was used. Flanking sequence positions with a probability of a single nucleotide lower than 0.3 were deleted. For Gcn4 and Vhr2, no apparent motifs were found in the promoter regions after the scan. To deal with this issue, one of the flanks of motif sequence positions probabilities was first evened out, and the software was rerun once again, searching for degenerative versions of the motif. If no motif was found in promoter regions after this, then iteratively, one of the flanks was deleted, and the other had its probabilities evened out (0.25, 0.25, 0.25, 0.25) then the software was rerun. The length of the motif was never smaller than 7bps. After obtaining the motif locations in the promoter regions, the signal of the relevant strains was extracted ±x bp from the center of the motif (100bp for Supplementary Figure S5, S10C and 50 bp in Figure 7D) and averaged to get the meta-motif profiles shown in Figures Supplementary Figure S5, S10C, and Figure 7D. The profile was smoothed with the average in rolling window of size 5 bps. The background in Supplementary Figures S5 and S10C is color-coded by the sum of the signal of the calculated meta-profile.

### Average binding at newly acquired minimal TF promoters

**Figure 3.**
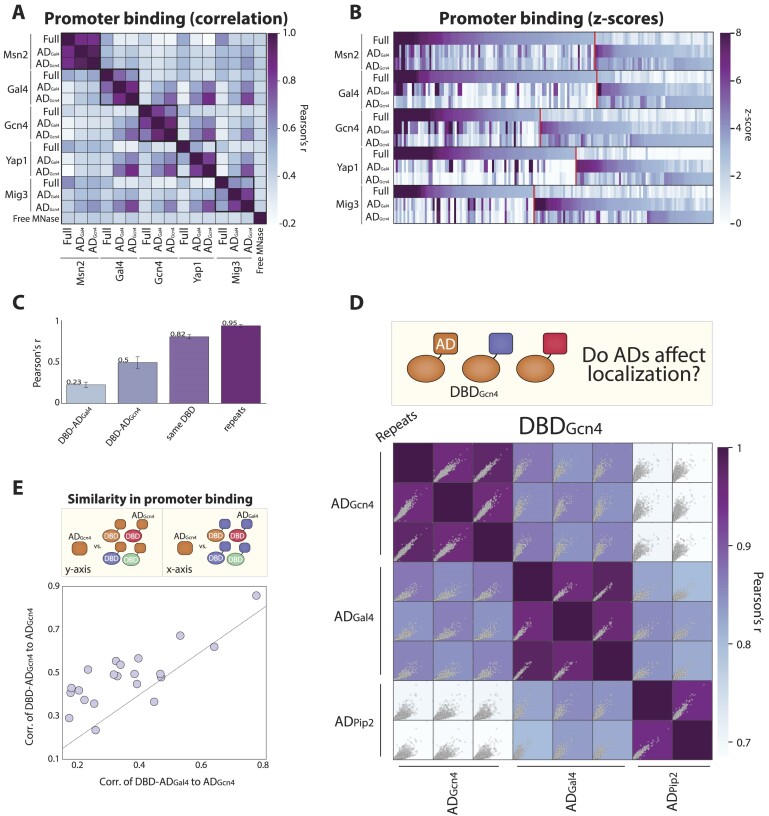
Binding preferences of minimal TFs follow the DBD but are also influenced by the fused AD. (A, B) Binding profiles of the minimal DBD-AD TFs differ from those of full TFs: shown in (**A**) is the similarity (correlation) of promoter binding preferences of the indicated TFs. Note the moderate correlation between most minimal and full TFs, the high correlation between minimal TFs carrying the same DBD, the higher correlation of minimal TFs carrying AD_Gcn4_ compared to those carrying AD_Gal4,_ and the low correlation of all presented TFs to nuclear-localized Free MNase. TF binding at individual promoters is shown in (**B**), with each column representing a promoter color-coded by the respective TF binding strengths. This figure includes all promoters bound by at least one of the indicated TFs. The red lines separate promoters defined as newly acquired by the DBD-ADs TFs (right, methods). (C–E) Binding profiles of minimal TFs depend on both the DBD and the AD: shown in (**C**) are the average correlation values of promoter selection between repeats, same-DBD TFs, and same-AD TFs. Note the high similarity between same-DBD minimal TFs and the moderate correlation between minimal TFs bearing AD_Gcn4_. Shown in (**D**) is the correlation of promoter preferences between minimal TFs containing DBD_Gcn4_, including technical repeats. Shown within each square is a direct comparison of promoter binding between each pair of samples, with each dot representing a promoter. (**E**) Shows the promoter selection correlation between AD_Gcn4_ to same-DBD TFs carrying AD_Gal4_ (x-axis) or AD_Gcn4_ (y-axis). Note the consistently higher correlation of AD_Gcn4_ carrying TFs.

**Figure 4.**
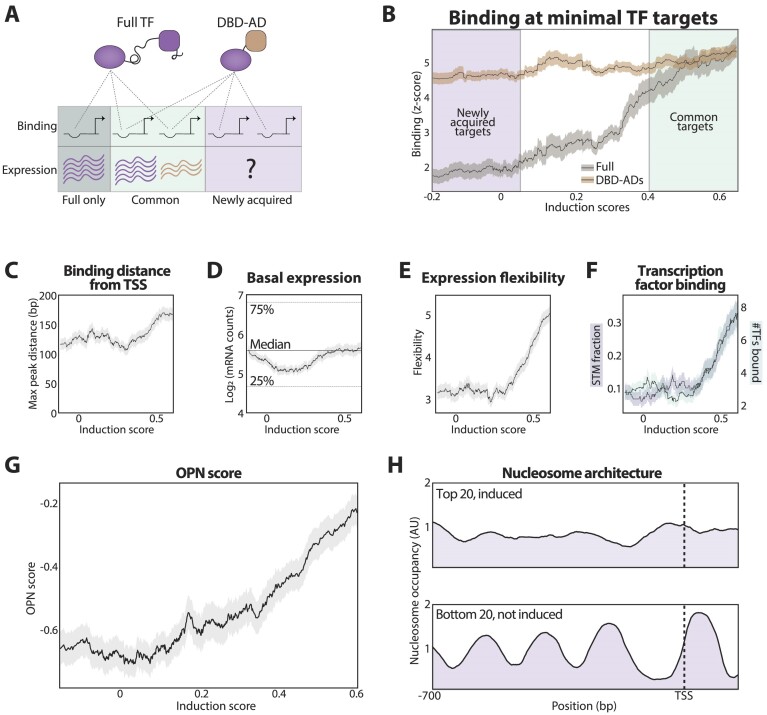
The transcription activity of DBD-AD fusions is limited to OPN-type promoters, showing flexible expression and binding by multiple TFs. (**A**) Experimental question: minimal DBD-AD TFs bind to promoters not bound by the full TFs. We asked whether they could induce gene expression at these newly acquired promoters. (**B**) DBD-AD factors show little activity at newly acquired promoters: shown is the average binding of minimal (brown) and full TFs (gray) at all minimal TF target promoters as a function of their averaged induction scores (calculated from the DBD-AD libraries, methods). The standard error of the mean (SEM) is shown as a shaded area for each line (methods). Note the low activation capacity at newly acquired DBD-AD promoters (left). (**C–G**) *Properties of promoters bound by minimal DBD-AD TFs*: Promoters were sorted by their gene induction score in the DBD-AD libraries. The averaged value of the indicated promoter properties is shown as a function of the averaged induction score (methods). The shaded area represents the SEM. Data for expression flexibility was taken from (60), and the data used for calculating the average fraction of STM (SAGA, Tup1, and Mediator) was taken from (61). Shown in (**H**) is the average nucleosome architecture as calculated from data obtained by MNase-seq (66) for the 20 most and 20 least induced gene promoters.

For Figure 4B, The z-score sum of signal over all newly acquired minimal TF promoters was calculated for all TFs presented in Figure 3A. Then, the combined vector of all binding z-scores of the full TFs (gray line) and all DBD-ADs (brown line) was sorted by the respective induction scores of the minimal TFs and averaged in a rolling window of 120. The error was calculated as the standard error of the mean (SEM) in the same window and shown as a background for each line.

### Binding distance from TSS

The ChEC-seq signal on target promoters of all minimal TFs was averaged by a rolling window of 50, and the distance of the maximal value of each signal from the TSS was extracted. The data was sorted by the respective induction score, averaged in the rolling window of the size of 150, and plotted in Figure 4C. The error was calculated as the standard error of the mean (SEM) in a same-sized window and shown as a colored background.

### OPN score calculation

**Figure 5.**
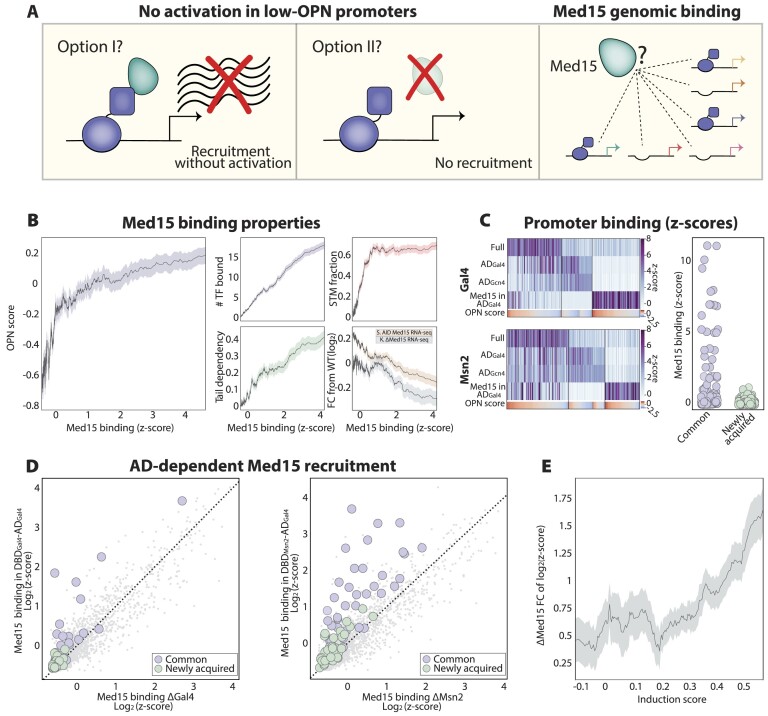
Med15 shows limited recruitment to low-OPN promoters. (**A**) Experimental question: the limited ability of minimal TFs to induce expression of low-OPN gene promoters may reflect the inactivity of Med15 recruited to those promoters or the inability of the localized AD to recruit Med15. We test this by measuring Med15 genomic localization in strains carrying minimal DBD-AD TF fusions. (**B**) In wild-type cells, Med15 preferentially localizes to high-OPN promoters displaying high flexibility and occupied by multiple TFs: Med15 binding profiles were measured in exponentially growing wild-type cells using ChEC-seq. The average promoter OPN score is shown as a function of the average Med15 binding strength (left). Note the high correspondence between Med15 binding data collected in this study and the average number of bound TFs (top left, methods), averaged fraction of genes previously defined as Mediator-tail-dependent (63) (bottom left), averaged fraction of promoters defined as bound by general coactivators (top right, STM) (61), and average expression changes upon Med15 depletion (65) (*S*)/deletion *(K)* (64) (bottom right; Supplementary Figure S12). (**C**) DBD-AD fusions fail to recruit Med15 to their newly acquired promoters: shown on the left is TF binding at individual promoters, as indicated. The columns represent promoters, ordered by their OPN scores (bottom panels, methods). The displayed promoters are bound by at least one of the presented TFs (z-score ≥ 3), and the black lines separate WT targets, unique minimal TF targets, and unique Med15 targets in cells expressing the indicated minimal TF. Shown on the right is the binding of Med15 to promoters bound by the minimal DBD-ADs TFs, distinguishing common promoters, also bound by the respective full TFs, and uniquely bound ones. Each point is a promoter, and all DBD-AD bound promoters are included (*z*-score ≥ 3). (**D**) DBD-AD fusions recruit Med15 to promoters bound by both minimal and full TFs: shown is the binding of Med15 to individual promoters in cells deleted of the respective native TF and expressing (y-axis) or lacking (x-axis) the respective DBD-AD fusion. Each dot is a promoter. Common targets of the minimal and full TFs and newly acquired targets of the minimal TFs are indicated by colors. (**E**) Med15 recruitment correlates with gene induction: Med15 recruitment was compared between TF-deleted cells containing or lacking respective DBD-AD fusions. The average change in Med15 binding is shown as a function of the average induction score. This analysis includes all DBD-AD bound promoters. The data is averaged by a rolling window of size 50, and the error was calculated as the standard error of the mean (SEM) in the same window and shown as a colored background.

**Figure 6.**
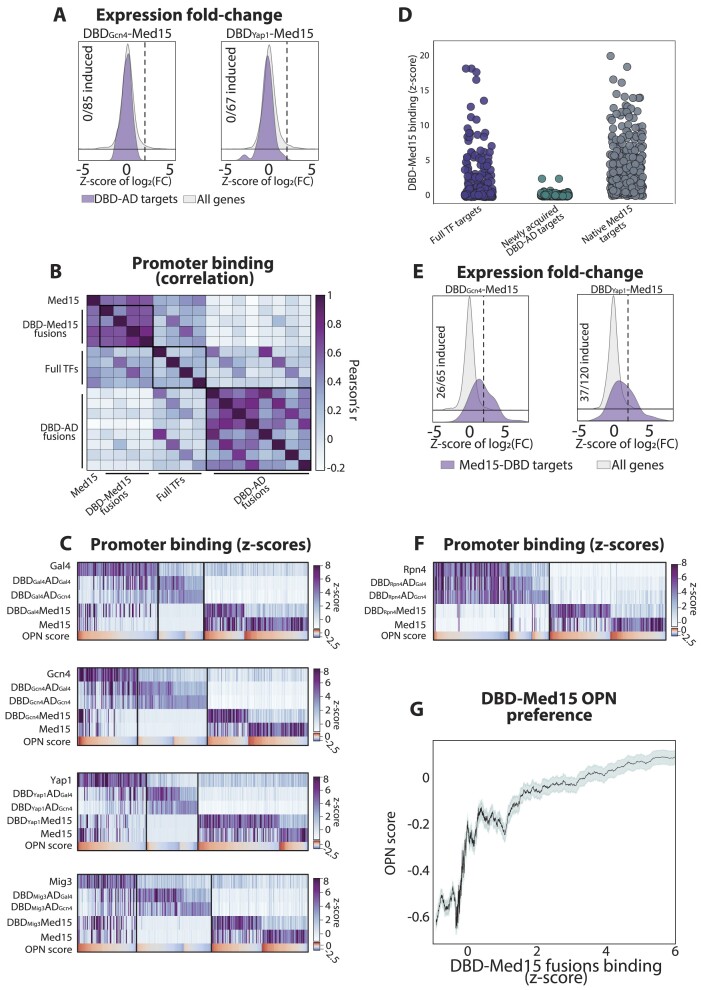
Med15 fusion redirects DBD binding towards promoters of fuzzy nucleosome architecture. (**A**) DBD-Med15 fusions fail to induce expression of newly acquired targets of the minimal TFs: cells expressing the two indicated DBD-Med15 fusions driven by the strong TEF1 promoter were engineered, and their genome-wide expression patterns were profiled. Shown are the distributions of expression changes, comparing genes bound by the respective DBD-AD (but not full) TFs to all other genes. (B–D) Med15 fusion redirects DBD bindings towards high-OPN promoters and away from minimal DBD-AD TF targets: binding profiles of the DBD-Med15 fusions were mapped. Shown in (**B**) are the correlations in promoter selection between the indicated sets of factors. Shown in (**C**) are the promoter binding strengths of the indicated TFs, as in Figure 5C. The binding signals of DBD-Med15 fusions at promoters bound by full TFs, unique DBD-ADs targets, and targets of the native Med15 are also shown (**D**, z-score ≥ 3). (**E**) DBD-Med15 fusions induce their bound targets: shown are the changes in expression of genes bound by the DBD-Med15 fusions (purple) as compared to all other genes (grey, methods). The dashed line indicates the threshold of significant gene-induction. The fraction of activated genes from the subset of bound ones is also indicated. (**F**) Med15 shifts the binding preferences of DBD_Rpn4_ towards high-OPN promoters: the same representation as in (C) for the DBD_Rpn4_-Med15 fusion. (**G**) Med15 fusion redirects DBD binding towards high-score OPN promoters: shown is the average OPN score as a function of the sorted and averaged binding strength of all DBD-Med15 fusions (methods).

OPN score was calculated as described in (59). The TSS proximal region was defined as the region from the TSS to 150 bp upstream of it. The TSS distal region was defined as the region between 200–400 bp upstream of the TSS. Then, the average nucleosome occupancy in the proximal and the distal regions was calculated, and the OPN-score was defined as log_2_(average nucleosome occupancy at distal TSS) – log_2_(average nucleosome occupancy at proximal TSS). For Figures 4G, 5B and 6G, the OPN score was sorted by the respective parameter presented on the x-axis of the plot and averaged by the rolling window of size 150, 150 and 500, respectively. Figure 6G presents data for all 5 Med15-DBD fusions in one plot. The error was calculated as the standard error of the mean (SEM) in the same window and shown as a colored background.

### Expression flexibility

The IDEA data was used to define the flexibility of each gene (60). This dataset includes the fold-change of each gene in S. cerevisiae across multiple data points following an induction of a specific TF (a total of 203 different TFs). For each gene, the flexibility was calculated as the difference between the log2 fold-change of percentile 0.05% to the log_2_ fold-change of percentile 99.95% over all time points of all TF time courses. The data was sorted by the induction score, averaged by a rolling window of size 150, and plotted in Figure 4D. The error was calculated as the standard error of the mean (SEM) in the same window and shown as a colored background.

### STM fraction calculation

The feature class 1 dataset from (61) was taken to calculate the STM fraction. Each gene marked by ‘02_STM’ was given a value of 1, and all other feature annotations were given a 0. To calculate the average ‘STM fraction’ shown in Figures 4F and 5B the data was sorted by the respective parameter presented on the x-axis of the plot, averaged by a rolling window of size 150, and plotted as a line. The error was calculated as the standard error of the mean (SEM) in the same window and shown as a colored background.

### The number of bound TFs

Data containing TF binding profiles for 145 TFs generated in our lab was used. The sum of the signal over each promoter was calculated for all TFs. Each TF sum on the promoter vector was z-score normalized. The number of TFs that bind a certain promoter with *z*-score ≥3 was assigned as the number of bound TFs for each promoter. The data was sorted by the respective parameter presented on the x-axis of Figures 4F and 5B, averaged by a rolling window of size 150. The error was calculated as the standard error of the mean (SEM) in the same window and shown as a colored background.

### Gene noise

Data from (62) was used to assign noise scores for each of the analyzed genes. The data was sorted by the induction score, averaged by a rolling window of size 150, and plotted as a line in Supplementary Figure S11. The error was calculated as the standard error of the mean (SEM) in the same window and shown as a colored background.

### Basal expression

The median of the log_2_ normalized data from RNA-seq of >60 BY4741 strains generated in the study was used to represent the ‘Basal expression level’ of each gene. The data was sorted by the induction score, averaged in the rolling window of size 150, and plotted in Figure 4D. The error was calculated as the standard error of the mean (SEM) in the same window and shown as a colored background. The defined Basal expression level of each gene was also used in Supplementary Figure S7B.

### Tail dependency

The data from (63) was used to define the fraction of Mediator tail-dependent genes. Each gene marked as ‘tail-dependent’ was given a value of 1, and all other feature annotations were given a 0. To calculate the average ‘Tail dependency’ fraction shown in Figures 4F and 5B the data was sorted by the respective parameter presented on the x-axis of the plot, averaged in the rolling window of size 150, and plotted as a line. The error was calculated as the standard error of the mean (SEM) in the same window and shown as a colored background.

### Analysis of Med15 deletion/depletion RNA-seq data

To validate the Med15 binding data generated in this study using the ChEC-seq method, data from (64,65) was used. Presented in Supplementary Figure S12A is data from (64). Here, we used the results of the deletion of the Mediator tail subunits (Med15, Med2 and Med3) and the deletion of subunits of the Mediator kinase module (Med13, Med12). The data was log_2_ normalized, and the control data presented in the study was subtracted. Next, the values were sorted by the Med15 binding *z*-scores obtained in this study, averaged by the rolling window of size 150, and plotted. In Figure 4B, data of deletion of Med15 (indicated by ‘K’) and data of Auxin Induced Degron (AID, indicated by ‘S’) obtained from (65) were averaged and presented in the same way. For all the presentations, the error was calculated as the standard error of the mean (SEM) in the same window and shown as a colored background.

### Genomic tracks normalization

**Figure 7.**
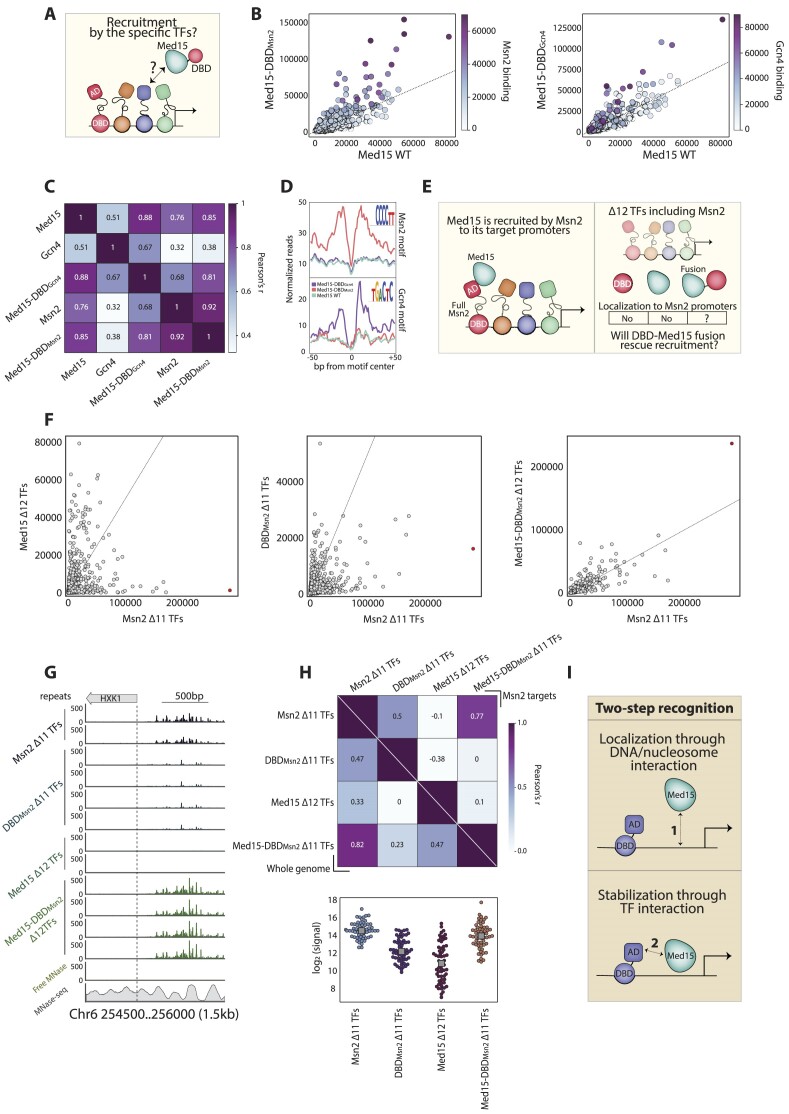
DBD-Med15 fusions gain new specific binding sites, including promoters unbound by the separated DBD and Med15. (**A**) Experimental question: we asked whether the binding preferences of the DBD-Med15 fusions are explained by their recruitment by specific TFs localizing to these promoters. (B–D) Med15-DBD fusions gain specific binding sites of the respective full TFs: we fused the endogenous Med15 to the DBDs of Msn2 and Gcn4 and profiled the genomic binding locations of the fused factors. Shown in (**B**) are the promoter binding signals, comparing Med15-DBD_Msn2_ (left) or Med15-DBD_Gcn4_ (right) to that of the endogenous Med15. Each dot is a promoter color-coded by the binding signal received for the respective full TF (Msn2 or Gcn4). Note the specific increase in Med15-DBD binding to promoters strongly bound by the corresponding full TFs. The correlations of promoter selection amongst all five profiles are also shown (**C**). As another indicator of the specificity of DBD-Med15 binding, we plotted the average signal around the DBD preferred motifs (**D**, methods). The seq-logo of the examined motif is shown on the right (methods). (E–H) *Med15 fusion can redirect DBD binding to promoters deprived of all Med15-recruiting TFs:* We considered a recently described strain (84) in which 12 stress-regulating TFs were deleted, including Msn2. In this 12-TF deleted mutant, Med15 binding is lost from Msn2 target promoters. The isolated DBD_Msn2_ does not bind these promoters either. We integrated the DBD_Msn2_-Med15 fusion into the genome of this 12-TF deleted strain and mapped its binding profile (**E**). Shown in (**F**) are scatterplots comparing promoter binding of the DBD_Msn2_ (left), Med15 (center) and the DBD_Msn2_-Med15 fusion (right), all measured in the 12-TF deleted strains to that of Msn2 in a strain deleted of the other 11 TFs. Note that Med15-DBD_Msn2_ retrieved binding to Msn2 target promoters. This retrieval of Msn2-like binding is further seen when examining the binding patterns upstream of individual genes (**G**, showing HXK1, marked by red in F). The bottom sub-panel indicates nucleosome occupancy measured by MNase-seq (66). Shown in (**H**, top) is the correlation of binding profiles across the genome (lower triangle) and at Msn2-induced targets (upper triangle), as defined in Figure 2C. The distribution of binding signal strengths of each of the indicated factors at Msn2-induced promoters is shown in (**H**, bottom). (**I**) *Proposed model*: We observe a strong Med15 preference for localizing to high-OPN promoters, manifested by the limited activity of ADs recruited to low-OPN ones and by its dominant influence on the binding upon its fusion to a DBD. We propose that Med15 localization is guided by interaction with nucleosomes or even with the DNA itself in a specific manner, thereby accounting for its differential recruitment across the genome. Following this localization, Med15 is stabilized by interaction with co-localizing TFs.

To present and compare the data across long genomic regions, as shown in Figures 7G, Supplementary Figures S1A, S3 and S4, we z-score normalized the signal across the entire genome for the indicated factors. To plot the average binding profiles at target promoters in Supplementary Figure S7A, counts per 10 million (CPtM) normalized reads for the set of target promoters were obtained for each respective TF and averaged. The same was also done for data obtained using MNase-seq collected from exponentially growing cells to represent the average state of nucleosome occupancy along the averaged meta-profile of target binding for each respective TF.

### Whole genome RNA-seq correlation

The log_2_ normalized data obtained by RNA-seq for the 15 TF promoter libraries was used to generate Supplementary Figure S2C. Each library was ordered by strain fluorescence, and then the median of each gene across all library vectors (>570 samples) was calculated and subtracted from each sample. Next, Pearson's r correlation coefficient between all these samples was calculated and shown as a heatmap.

### Nucleosome meta profile

To plot the meta nucleosome profiles shown in Figure 4H, we used previously published MNase-seq data collected from exponentially growing cells (66). Following read normalization to 10e6, the signal received on the 20 promoters with the highest and the 20 with the lowest induction scores of all minimal TF libraries was averaged and plotted.

## Results

### Designing minimal TFs for directing ADs to hundreds of promoters

Minimal TFs, consisting of a DBD-AD fusion, can be used to compare AD activity and its potential influence on binding across different regulatory regions (Figure 1A). To test this, we set to generate DBD-AD fusions that will localize to a wide range of promoters. Previous studies revealed that TF deletion or over-expression often leads to limited expression changes (60,64,67, likely reflecting condition-specific inhibition of DNA binding or transcription activity. As these effects could also limit the function of our minimal TFs, we conducted a prescreen to select DBD-AD combinations of detectable activity.

We selected 16 DBDs from TFs that bind across a broad range of promoters, as seen in our TF-binding compendium (66,68–70), and further chose the two well-characterized ADs of Gcn4 and Gal4 (Figure 1B, C, Supplementary Figure S1A, B). Both ADs are relatively short and occupy a small portion of the respective TF sequences (43/281 and 49/881 AA). Of note, both ADs interact with the same coactivator, Med15, a component of the Mediator tail subunit (8,10,71,72). We favored the Gal4 AD_2_ (C-terminal AD) despite its known interaction with the Gal80 inhibitor, as we expected this inhibition to be readily saturated in our system upon over-expression.

We engineered the 32 DBD-AD combinations and integrated them and the 16 full TFs into the genome downstream to a strong promoter (TEF1) while adding a nuclear localization signal (NLS, Figure 1C, Supplementary Figure S1C). We then profiled gene expression of the 44/48 successfully engineered strains (Figure 1D, E) and analyzed their effect on genes bound by the natively expressed full TFs (Figure 1B). As expected, the overexpression of most TFs had limited consequences on gene expression (Figure 1F). For our subsequent analysis, we, therefore, selected the five DBD-TF sets that did show considerable transcription effects, including three activators (Gal4, Gcn4 and Msn2), one repressor (Mig3), and one that both induced and repressed its targets (Yap1).

### Library-based approach for measuring the genome-scale transcription activities of full and minimal DBD-AD TFs

Asensitive comparison of transcriptional effects exerted by a TF requires the control of its abundance. To enable that, we devised a library-based approach, in which we generated 35–40 strains, each expressing the TF of interest at a different level, driven by a set of synthetic promoters as quantified by YFP-fusion (Figure 2A). Focusing on the five DBD sets selected in our prescreen, we generated fifteen libraries, including the full and two minimal TFs, and mapped the genome-wide expression profiles of all 570 strains.

Increasing TF abundance gradually induced the expression of its known target genes (Figure 2B, Supplementary Figure S2A). To quantify the TF-gene effect with a single number, we calculated the correlation between the abundance of the TF and the expression levels of each gene and defined this as the ‘gene induction score’. In each library, we then defined the subset of induced targets as the set of promoters that received high induction scores and were bound by the respective native TF (methods, Figure 2C; Supplementary Figure S2B). The subsets of induced targets were similar between most of the same-DBD factors (e.g. Gcn4; Figure 2D; Supplementary Figure S2B, C) but differed somewhat in others (e.g. Gal4). Mig3 displayed some positive correlation with Gcn4, pointing perhaps to indirect effects (Figure 2D, Supplementary Figure S2B, C).

In most cases, the full TFs induced a larger fraction of targets and reached higher induction levels than the respective DBD-AD fusions (Figure 2E, F; Supplementary Figure S2D). The full Msn2, for example, induced ∼70% of the native Msn2 bound targets to an average of 3.8 log2 folds, while the respective DBD_Msn2_-AD_Gal4_ and DBD_Msn2_-AD_Gcn4_ induced only 54.5% and 36.4% of these same promoters, to an average of 3.38 and 1.85 log_2_-folds respectively. The DBD_Gcn4_-AD_Gcn4_ was an exception, reaching similar induction levels to those of the full Gcn4. The full Mig3 downregulated its target genes, consistent with its role as a transcription repressor. We conclude that the library-based analysis provides a sensitive measure of TF activity on individual gene promoters.

### The minimal DBD-AD TFs acquire new promoters, dominated by the DBD but influenced also by the fused AD

Above, we used our library-based approach to examine the transcription induction of genes bound by the endogenous TFs. However, the genomic binding of isolated DBDs often differs from those of full TFs (29,70), which could explain the weaker transcriptional effects of the DBD-AD factors when measured on these promoters. To test that, we mapped the genomic binding of the over-expressed minimal and full TFs in cells deleted of the respective native TF using ChEC-seq (73) (Supplementary Figure S1C; see Supplementary Figure S3–S5). As expected, binding profiles differed between minimal and full TFs (Figure 3A; Supplementary Figures S3B, S4), and following experiments with added calibration controls further verified the predicted weaker binding of the DBD-AD fusions at targets of the native TFs, consistent with their weaker transcription effects (Supplementary Figure S6) (29,70).

The DBD-ADs localized to new promoters not bound by the full TFs (Figure 3B; Supplementary Figure S7). Those newly acquired promoters were enriched with the DBD-preferred motifs and correlated well with binding of DBD-only mutants expressed under the native TF promoters (29,70) (Figure 3A,B; Supplementary Figures S8 and S9A). Notably, while the DBD identity dominated promoter selection, the fused AD also contributed to the genomic localization pattern (Figure 3C). Thus, the similarity in promoter binding of same-DBD factors was high (average Pearson's *rc* = 0.82 ± 0.02) but still lower than between technical repeats (Pearson's *rc* = 0.95 ± 0.015). Further, minimal TFs carrying AD_Gcn4_ were more correlated amongst themselves than factors carrying AD_Gal4_ (Pearson's *rc* = 0.5 ± 0.07 compared to *c* = 0.23 ± 0.03).

To further verify the influence of ADs on binding preferences, we fused additional ADs to the DBDs of Msn2 and Gcn4. In the case of Msn2, the binding profiles of the ten DBD-AD fusions were similar, all located in the range between the full Msn2 and the DBD-only mutant (Supplementary Figure S9B). By contrast, in the case of Gcn4, the three successfully profiled minimal TFs were all quite different, pointing to a considerable influence of the AD identity (Figure 3D). In this case, we could not obtain reproducible profiles for the isolated DBD and 7/10 DBD-AD fusions, all of which were slow growers. Finally, we also obtained reproducible profiles for the isolated AD of Gcn4 (but not of Gal4). Comparing its binding preferences to those of our DBD-AD fusions (including those used in our prescreen) verified its higher similarity to the minimal TF carrying the Gcn4 AD (Figure 3E; Supplementary Figure S10A–D). Together, we conclude that the DBDs dominate the genomic localization preferences of minimal TFs, with the fused ADs providing an additional, unexpected contribution.

### The minimal DBD-AD TFs induce gene expression from only a subset of bound promoters, characterized by fuzzy nucleosome architecture

The minimal TFs showed similar binding at promoters that did or did not bind the respective full TFs (Figure 3B). We, therefore, expected the transcription activity of the DBD-AD factors to be similar between these sets of common and newly acquired promoters, namely those that were not bound by the full TFs (but often bound by DBD-only mutants Figure 4A and Supplementary Figure S9A). This, however, was not the case, as newly acquired promoters showed little if any, gene induction (Figure 4B). This was also true for most DBD-AD tested in our prescreen, with two notable exceptions (DBD_Sko1_-AD_Gcn4_ and DBD_Cst6_-AD_Gcn4_; Supplementary Figure S10E).

The minimal TFs, therefore, induced expression only from a subset of their bound promoters, corresponding primarily to those also bound by the full TFs. To define the properties distinguishing the induced promoters, we tested for promoter features that correlate with the gene-induction scores, as defined above. On average, the binding of the minimal TFs at induced promoters was somewhat further from the TSS as compared to uninduced promoters (Figure 4C), and their steady-state expression was a bit higher (Figure 4D). Larger differences were observed in expression flexibility, with induced promoters showing higher responsiveness to various TFs (60), and higher variability between individual cells (noise, Figure 4E, and Supplementary Figure S11) (62). Those promoters were also more likely to bind multiple specific TFs, as defined in a recent ChIP-Exo analysis (61) and our lab database (Figure 4F).

Gene expression flexibility correlates with the architecture of promoter nucleosomes, as captured by the so-called OPN score (for ‘Occupied Proximal Nucleosome’), measuring the fuzziness of nucleosome occupancy (59). Indeed, the induction scores correlated tightly with the promoter OPN score, with little induction seen in low-OPN promoters (Figure 4G, H). Therefore, the capacity of promoter-bound ADs to induce gene expression correlates with the promoter nucleosome architecture.

### The minimal DBD-AD TFs recruit Med15 to only a subset of their bound promoters, characterized by fuzzy nucleosome architecture

Activation domains induce gene expression by recruiting coactivators. The failure of the DBD-AD to induce gene expression from a large fraction of their bound promoters may result from the limited activity of the recruited coactivator or from limited recruitment of the coactivators themselves to these loci (Figure 5A). Both ADs used here induce gene expression by recruiting the Mediator complex through interaction with Med15, a component of the Meditator tail subunit. We, therefore, asked whether the DBD-AD fusions recruit Med15 to all or only the activated subset of their bound promoters.

To test this, we used ChEC-seq to map the genomic localization of Med15 in wild-type cells and in cells expressing the minimal DBD-AD TFs. The Med15 profile in wild-type cells was consistent with preferences previously described using ChIP-seq and with studies defining the identities of genes dependent on the tail subunit of the Mediator complex (63–65,74) (Figure 5B; Supplementary Figure S12). The apparent absence of the Mediator from most other promoters is explained by its transient, tail-independent recruitment, revealed only upon stabilizing by kin28 depletion (63,74–80).

Promoters bound by Med15 in wild-type cells were almost exclusively of high OPN scores and were occupied by multiple specific TFs. By contrast, the newly acquired DBD-AD promoters were mostly of low OPN scores. Testing the Med15 genomic localization in cells expressing the minimal DBD-AD fusions revealed that it was virtually absent from the newly acquired minimal TF promoters (Figure 5C; Supplementary Figure S13). In fact, the DBD-AD fusions increased Med15 binding in only a subset of their bound promoters, which corresponded to the transcriptionally induced ones, which, as mentioned above, were also bound by the full TFs (Figure 5D, E). Therefore, the minimal DBD-AD TFs recruit Med15 to only a subset of promoters, characterized by high OPN scores.

### Med15 fusion redirects DBDs towards high-OPN promoters

The DBD-AD, therefore, can efficiently recruit Med15 and induce expression from some of their bound promoters but fail to do so in others. To further understand this limited activity of the DBD-AD in this latter promoter class, we next asked whether we can drive their expression by forcing Med15 recruitment. Motivated by previous studies showing that direct DBD-Med15 fusion strongly induces the expression of individual reporters (7,81–83), we fused Med15 to the DBDs of Gcn4 and Yap1 and tested the resulting whole-genome expression changes. Neither fusion, however, has led to increased expression from the newly acquired DBD-AD promoters (Figure 6A).

Given the lack of transcription induction of the newly acquired minimal TF targets, we asked whether the DBD-Med15 fusions bind these promoters. For this, we generated two additional fusions and mapped the binding profiles of the four Med15-fused factors (Figure 6B). Notably, the binding of all four was restricted to promoters of high OPN scores while being largely absent from the newly acquired promoters bound by the DBD-ADs (Figure 6C, D; Supplementary Figure S14A). Gene expression profiles further verified strong induction of the bound promoters (Figure 6E). As a further test of the Med15 ability to shift binding towards OPN promoters, we considered Rpn4, as both the full Rpn4 and its DBD-related minimal TFs distinctively bind at low-OPN promoters (Supplementary Figures S10A, S14B). Also, in this case, Med15 fusion shifted the binding of the Rpn4 DBD to high-OPN promoters (Figure 6F). We conclude that the Med15 redirects the binding of fused DBDs to promoters of fuzzy nucleosome architecture (Figure 6G).

### Med15-directed DBD binding is independent of interactions with promoter-localized TFs

Our results above revealed that Med15 redirects the genomic binding of fused DBDs to promoters of high OPN scores. As multiple specific TFs also localize to these promoters, we reasoned that they could redirect DBD-Med15 binding as they recruit the endogenous Med15 (Figure 7A). This explanation, however, faced several difficulties. First, the DBD-Med15 fusions in our system were overexpressed at levels that greatly surpassed the endogenous Mediator or specific TFs. Second, the (over-expressed) DBD-Med15 acquired binding at new promoters lacking the endogenous Med15 (Supplementary Figure S15A, B). In addition, the selection of bound promoters was also influenced by the DBD, as each DBD-Med15 fusion binding profile was biased towards targets of the respective full TF (Figure 6B; Supplementary Figure S15B). Still, almost no newly bound promoters overlapped those of low OPN scores, favored by the DBD-AD (Figure 6B–D, F).

Given these results, we hypothesized that Med15 fusion redirects DBD binding independently of promoter-localizing TFs, which generally recruit the endogenous Mediator. To test this prediction, we first verified that Med15 can redirect DBD binding when expressed at endogenous levels. Indeed, fusing the DBDs of Msn2 and Gcn4 directly to the endogenous Med15 has redirected the DBDs for binding OPN promoters, favoring targets of the respective full TFs and localizing to the DBD-preferred motifs (Figure 7B–D; Supplementary Figure S15C).

Next, we established a setting in which Med15-bound promoters are depleted of all Med15-recruiting TFs and, therefore, lose Med15 binding. In wild-type cells, Med15 is recruited to high OPN, Msn2/4 bound stress promoters, to kickstart gene production (80). To disrupt this, we used a strain in which we deleted Msn2/4 and ten additional TFs co-localizing to these promoters (84). Neither the Msn2 DBD nor Med15 can bind these promoters in the 12 TF-deleted strain (Figure 7E, F). Notably, as predicted, the Med15-DBD_Msn2_ fusion regained binding to these TF-depleted promoters (Figure 7F–H). We conclude that Med15 redirects the binding of the Msn2 DBD independently of its promoter-recruiting TFs, suggesting it possesses an inherent preference for binding specific promoters.

## Discussion

The regulation of gene expression depends on interactions between specific TFs and general coactivators such as Med15. Coactivators lack annotated DBDs and, when tested outside cells, interact with TF ADs in the absence of DNA. It was, therefore, assumed that ADs and coactivators lack inherent DNA preferences. In this simplified, linear view, a TF first binds to specific regulatory regions and subsequently recruits coactivators to those bound locations. Under this assumption, the fact that TFs often induce only a subset of their bound promoters is attributed to post-recruitment effects (85,86). This was shown, for example, for Gcn4 binding at open-reading frames, which did recruit the SAGA and SWI/SNF complex but did not lead to productive transcription (87). In this context, the most revealing aspect of our study is that ADs and Med15 can redirect the binding of DBDs. This was most notable for Med15, which strongly shifted preferences of fused DBDs towards a new set of promoters characterized by fuzzy nucleosome architecture.

Since we chose for our study ADs that recruit the Mediator complex by interacting with the component of its tail subunit Med15, we expected that fusing DBDs to Med15-interacting ADs would mimic direct DBD-Med15 fusions. This, however, was not the case, as the binding profiles of these two fusions showed major differences. The DBD-ADs have reduced their preference for binding promoters of fuzzy nucleosome architecture (high-OPN) and were bound accordingly to many low-OPN promoters, similar to the isolated DBDs. Notably, direct Med15 fusion shifted DBD binding away from low-OPN and towards high-OPN promoters. Consistently, the minimal DBD-AD TFs only recruited Med15 to high-OPN promoters but not to the low-OPN ones, and only these genes with fuzzy nucleosome promoters were transcriptionally activated by the DBD-AD or the DBD-Med15 fusions.

Fuzzy-nucleosome promoters, favored by Med15, are characterized by the binding of multiple Med15 recruiting TFs. These TFs are not, in fact, required for the binding of the Med15-DBD fusions, as Med15 was still capable of redirecting the Msn2 DBD to such promoters even in their absence. Med15, therefore, can recognize these promoters independently of bound TFs. The basis of this recognition is not yet clear. It could result from the interaction of the Med15-DBD with other Mediator or PIC components if these show TF-independent localization to the tested promoters. In this case, the lack of the native Med15 from these promoters is explained by the need for additional stabilization provided by the DBD. Alternatively, Med15 could more directly recognize the DNA or chromatin architecture of its favored promoters.

Our results add a new, seemingly critical, specificity-conferring factor to the current model of PIC formation. In budding yeast, PIC formation follows two distinct paths. The first is seen in core promoters that are devoid of specific TFs. In these promoters, the tail subunit is dispensable, and the Mediator binds only transiently to complete the assembly of the PIC (74,75,77,80). We now find that recruiting ADs to these TF-lacking promoters using various DBDs is not sufficient for stabilizing Med15 binding or inducing further transcription. The second path of PIC formation is seen at UASs of specific TF-regulated promoters. Here, stable recruitment of the Mediator tail is the required first step for Mediator binding and PIC formation (80). Our central result is that Med15 binding at these promoters cannot be explained as mere passive recruitment by promoter-localized TFs. We conclude that Med15 displays an inherent preference for these promoters and thereby actively contributes to the selection of induced genes and, perhaps, to the speed of target activation.

Based on our results, we propose that Med15 localizes to its target promoters through a two-step mechanism. First, Med15 recognizes specific promoters by either associating with components of the general machinery or, as we favor, through fuzzy and weak interactions dictated by specific DNA sequences or shapes. Second, TFs bound to these promoters stabilize and orient Med15 binding through AD-based Med15 interaction (Figure 7I). Perhaps supporting this, a recent structural study of the Med-PIC complex formed on a divergent promoter revealed a direct interaction between DNA and ∼100 AA region of Med15 (88), potentially refuting the long-standing assumption that Med15 lacks a DBD (36). Further studies are required to distinguish between those possibilities.

## Supplementary Material

gkae718_Supplemental_Files

## Data Availability

All data collected in this study is found in: https://www.ncbi.nlm.nih.gov/geo/query/acc.cgi?acc=GSE234433. The link to the code and notebooks to produce all of the figures: https://doi.org/10.5281/zenodo.12684310.
