# Supplementary material for: Revisiting the model for coactivator recruitment: Med15 can select its target sites independent of promoter-bound transcription factors
Source: Nucleic Acids Res. 2024 Aug 27;52(20):12093–111. doi: 10.1093/nar/gkae718 (PMC11551773; doi:10.1093/nar/gkae718)
Supplement: gkae718_Supplemental_Files [file gkae718_supplemental_files.zip › Supp-merged.pdf]

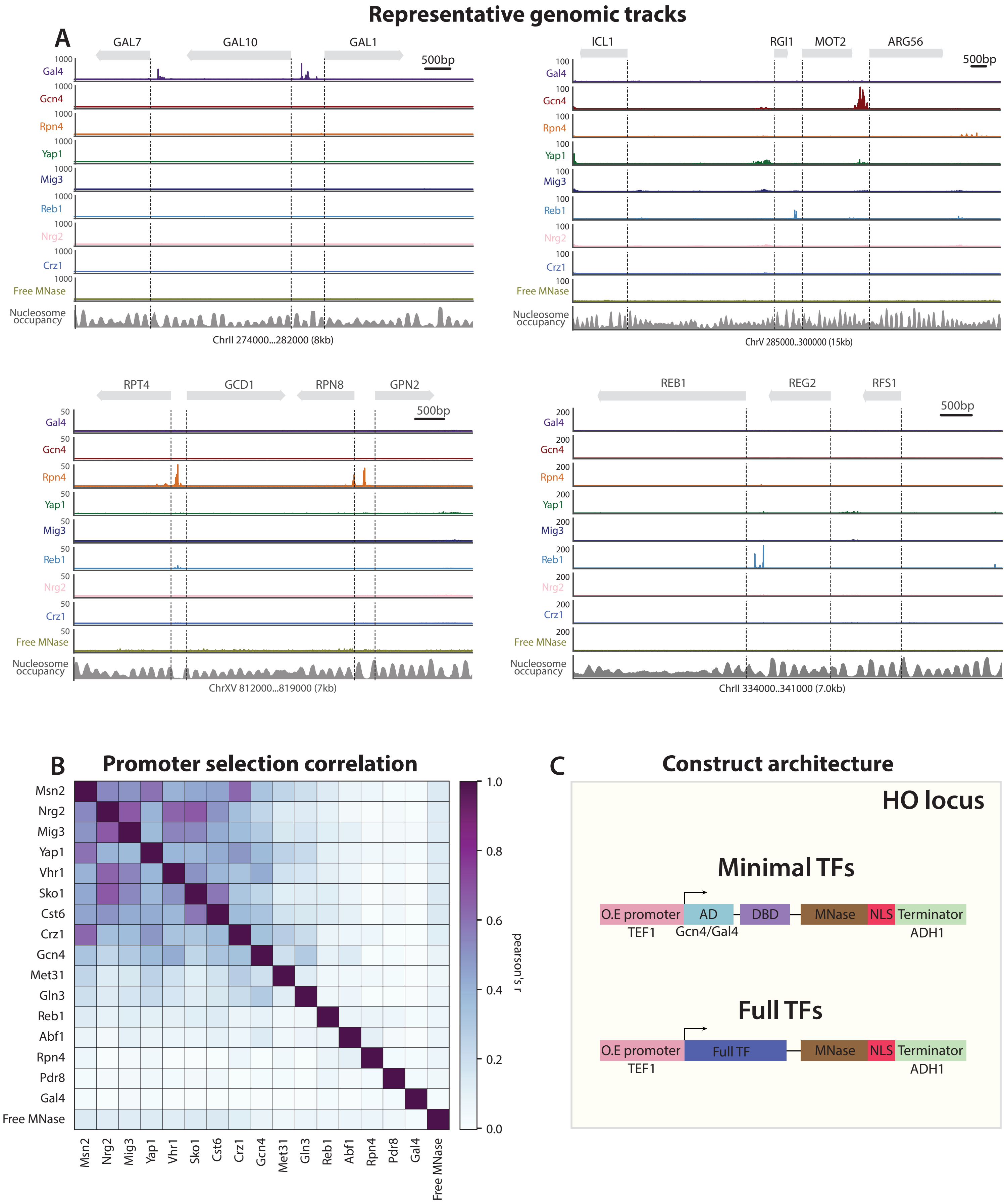

Representative genomic tracks
A
GAL7
GAL10
GAL1
500bp
Gal4
Gcn4
Rpn4
Yap1
Mig3
Reb1
Nrg2
Crz1
Free MNase
Nucleosome occupancy
ChrII 274000...282000 (8kb)
ICL1
RGI1
MOT2
ARG56
ChrV 285000..300000 (15kb)
RPT4
GCD1
RPN8
GPN2
ChrXV 812000...819000 (7kb)
REB1
REG2
RFS1
ChrII 334000..341000 (7.0kb)
B
Promoter selection correlation
Msn2
Nrg2
Mig3
Yap1
Vhr1
Sko1
Cst6
Crz1
Gcn4
Met31
Gln3
Reb1
Abf1
Rpn4
Pdr8
Gal4
Free MNase
pearson's r
1.0
0.8
0.6
0.4
0.2
0.0
C
Construct architecture
HO locus
Minimal TFs
O.E promoter
AD
DBD
MNase
NLS
Terminator
TEF1
Gcn4/Gal4
ADH1
Full TFs
Full TF

Figure S1. TFs from which DBDs were taken for our pre-screen and construct architecture:

(A-B) *Selected TFs localize to diverse genomic loci:* Shown in (A) are representative genomic tracks of known target genes of Gal4 (top left), Gcn4 (top right), Rpn4 (bottom left), and Reb1 (bottom right, methods). The sum of the signal over each promoter in the genome was calculated, and the correlation (Pearson's r) of promoter selection between the chosen TFs and a nuclear-localized MNase is shown in (B, methods).

(C) *Construct scheme:* Our construct was inserted into the HO locus (methods). Both the minimal and full TFs were fused to an MNase and over-expressed under the strong TEF1 promoter. The factors were also fused to an SV40 NLS.

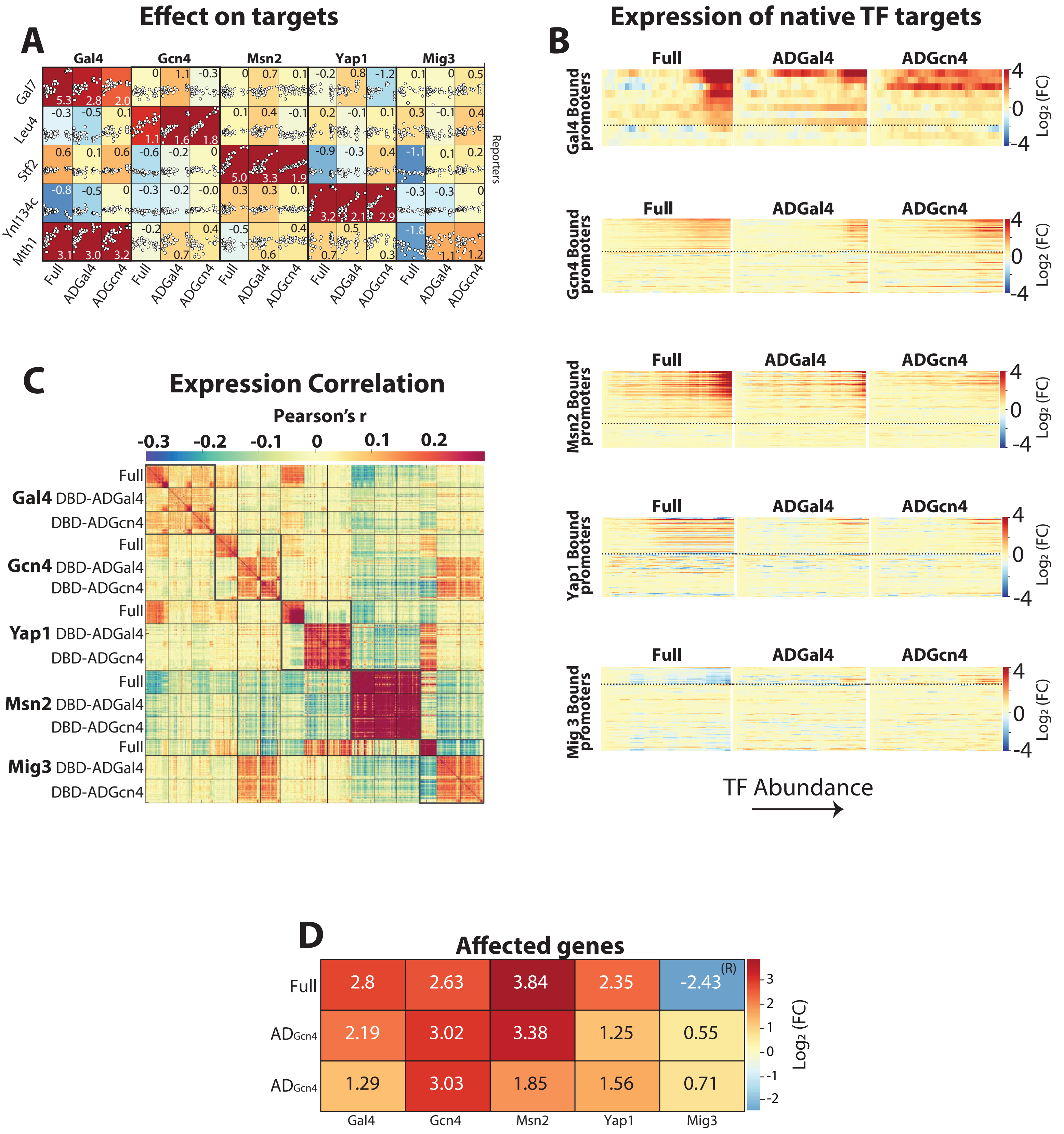

A
Effect on targets
Gal4
Gcn4
Msn2
Yap1
Mig3
Gal7
Leu4
Stf2
Ynl134c
Mth1
Full
ADGal4
ADGcn4
Reporters
B
Expression of native TF targets
Full
ADGal4
ADGcn4
Gal4 Bound promoters
Gcn4 Bound promoters
Msn2 Bound promoters
Yap1 Bound promoters
Mig 3 Bound promoters
Log2 (FC)
TF Abundance
C
Expression Correlation
Pearson's r
-0.3
-0.2
-0.1
0
0.1
0.2
Gal4 DBD-ADGal4
Gcn4 DBD-ADGal4
Yap1 DBD-ADGal4
Msn2 DBD-ADGal4
Mig3 DBD-ADGal4
DBD-ADGcn4
D
Affected genes
Full 2.8 2.63 3.84 2.35 -2.43 (R)
ADGcn4 2.19 3.02 3.38 1.25 0.55
ADGcn4 1.29 3.03 1.85 1.56 0.71
Gal4 Gcn4 Msn2 Yap1 Mig3
Log2 (FC)

Figure S2. Synthetic promoter libraries allow sensitive measuring of target gene expression:

(A-D) *Reporter genes show gradual expression change with increasing TF abundance:* Shown in (A, grey dots) for all minimal and full TFs is the effect of their increasing abundance (x-axis) on the expression levels of selected target genes (y-axis), where each dot represents a library strain. The log2 fold-change between the average target expression level in the 5 highest and the 5 lowest abundant strains is indicated on the bottom right of each square. Shown in (B) are the expression levels of all native TF-bound genes in each strain of each library (methods). Strains are ordered based on TF abundance (columns), and genes are ordered based on the fold change within the respective full TF library (rows). The black line separates genes passing the induction score threshold in the respective full TF library (methods). The median-normalized whole-genome gene expression correlation across the indicated libraries is presented in (C, methods). Strains within each library are ordered based on TF abundance, as measured by FACS. The average log2 Fold Change (FC) of native TF-bound targets that passed the induction score threshold is shown in (D). Note that for full Mig3, we present repression values consistent with its role as a repressor.

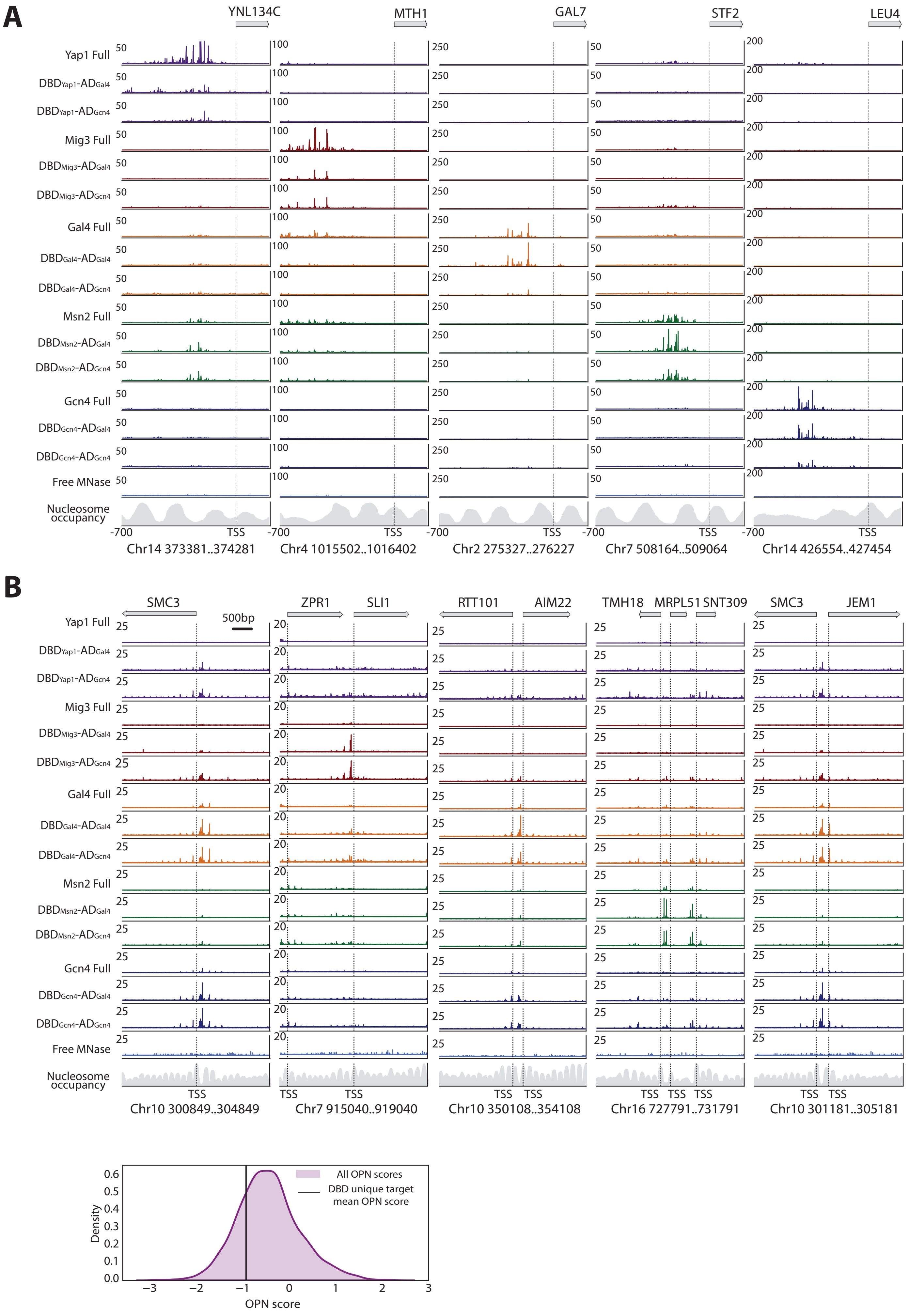

A
YNL134C
MTH1
GAL7
STF2
LEU4
Yap1 Full
DBDYap1-ADGal4
DBDYap1-ADGcn4
Mig3 Full
DBDMig3-ADGal4
DBDMig3-ADGcn4
Gal4 Full
DBDGal4-ADGal4
DBDGal4-ADGcn4
Msn2 Full
DBDMsn2-ADGal4
DBDMsn2-ADGcn4
Gcn4 Full
DBDGcn4-ADGal4
DBDGcn4-ADGcn4
Free MNase
Nucleosome occupancy
-700
TSS
Chr14 373381..374281
Chr4 1015502..1016402
Chr2 275327..276227
Chr7 508164..509064
Chr14 426554..427454
B
SMC3
500bp
ZPR1
SLI1
RTT101
AIM22
TMH18
MRPL51
SNT309
JEM1
Chr10 300849..304849
Chr7 915040..919040
Chr10 350108..354108
Chr16 727791..731791
Chr10 301181..305181
All OPN scores
DBD unique target mean OPN score
Density
OPN score

Figure S3. Raw data comparing minimal and full TFs:

(A) *Binding signals correspond to expression profiles:* Shown are the binding signals of the indicated TFs on the five gene promoters presented in (Figure S2. A, methods). The nucleosome occupancy captured by MNase-seq is also shown [1]. Note the high correspondence between the binding signal and the expression effect measured for the corresponding TF libraries.

(B) *Minimal TFs occupy low OPN score promoters not bound by the full TFs*: Shown are the binding signals of 5 low-OPN score target promoters of the DBD-AD fusions (presentation as in A); Shown at the bottom-left is a density plot of OPN scores for all analyzed promoters. A black line denotes the mean OPN score of the 5 promoters presented above.

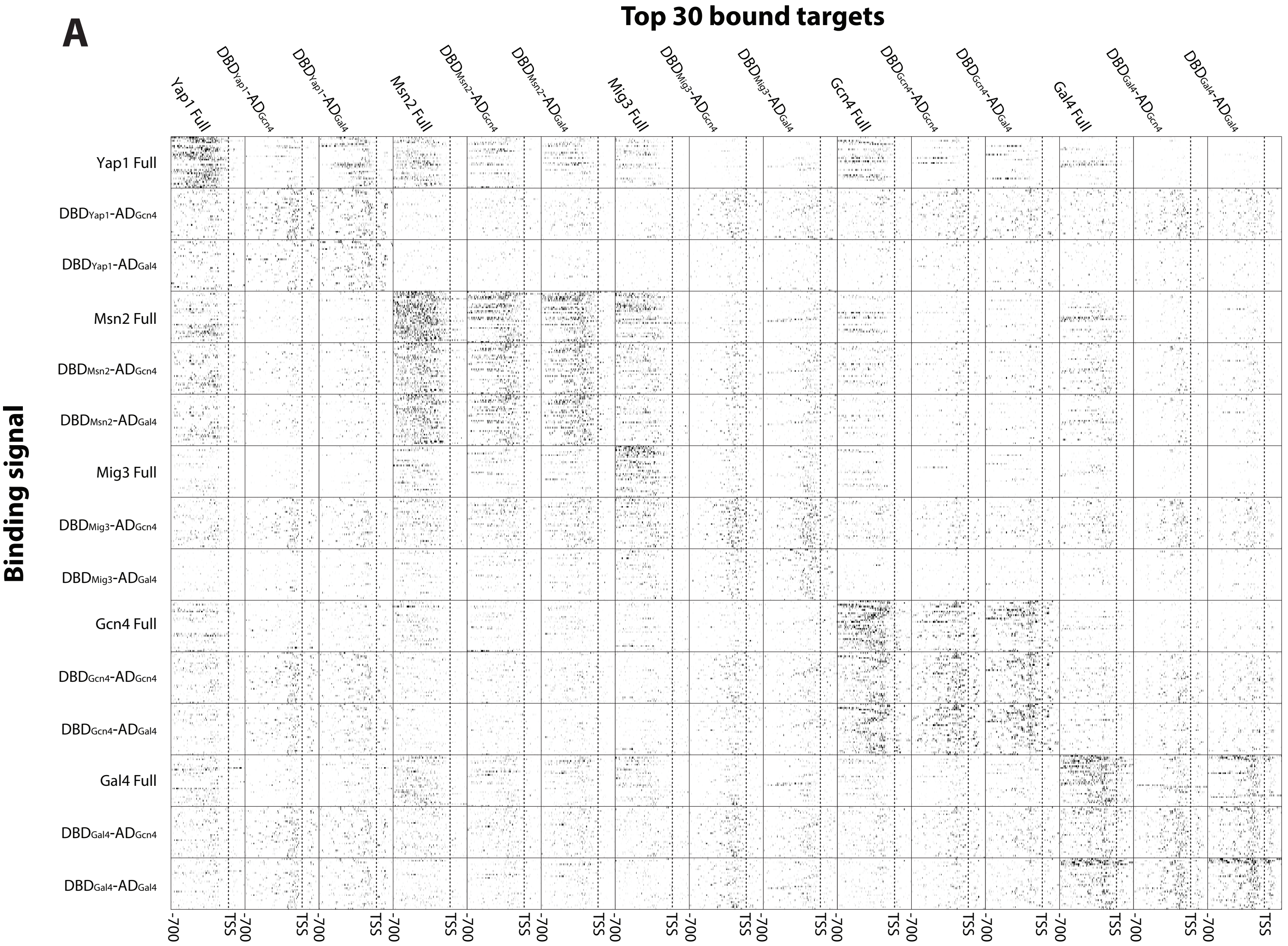
A
Top 30 bound targets
Binding signal
Yap1 Full
DBDYap1-ADGcn4
DBDYap1-ADGal4
Msn2 Full
DBDMsn2-ADGcn4
DBDMsn2-ADGal4
Mig3 Full
DBDMig3-ADGcn4
DBDMig3-ADGal4
Gcn4 Full
DBDGcn4-ADGcn4
DBDGcn4-ADGal4
Gal4 Full
DBDGal4-ADGcn4
DBDGal4-ADGal4
-700
TSS

Figure S4. Comparison of binding between minimal and full TFs:

(A) *Minimal TFs localize both to the targets of the corresponding full TF and to new targets:* Each square shows the signal (methods) received for the TF indicated on the left across the 30 strongest bound promoters of the TF indicated on the top. Each row is a promoter, and the dashed line marks the TSS.

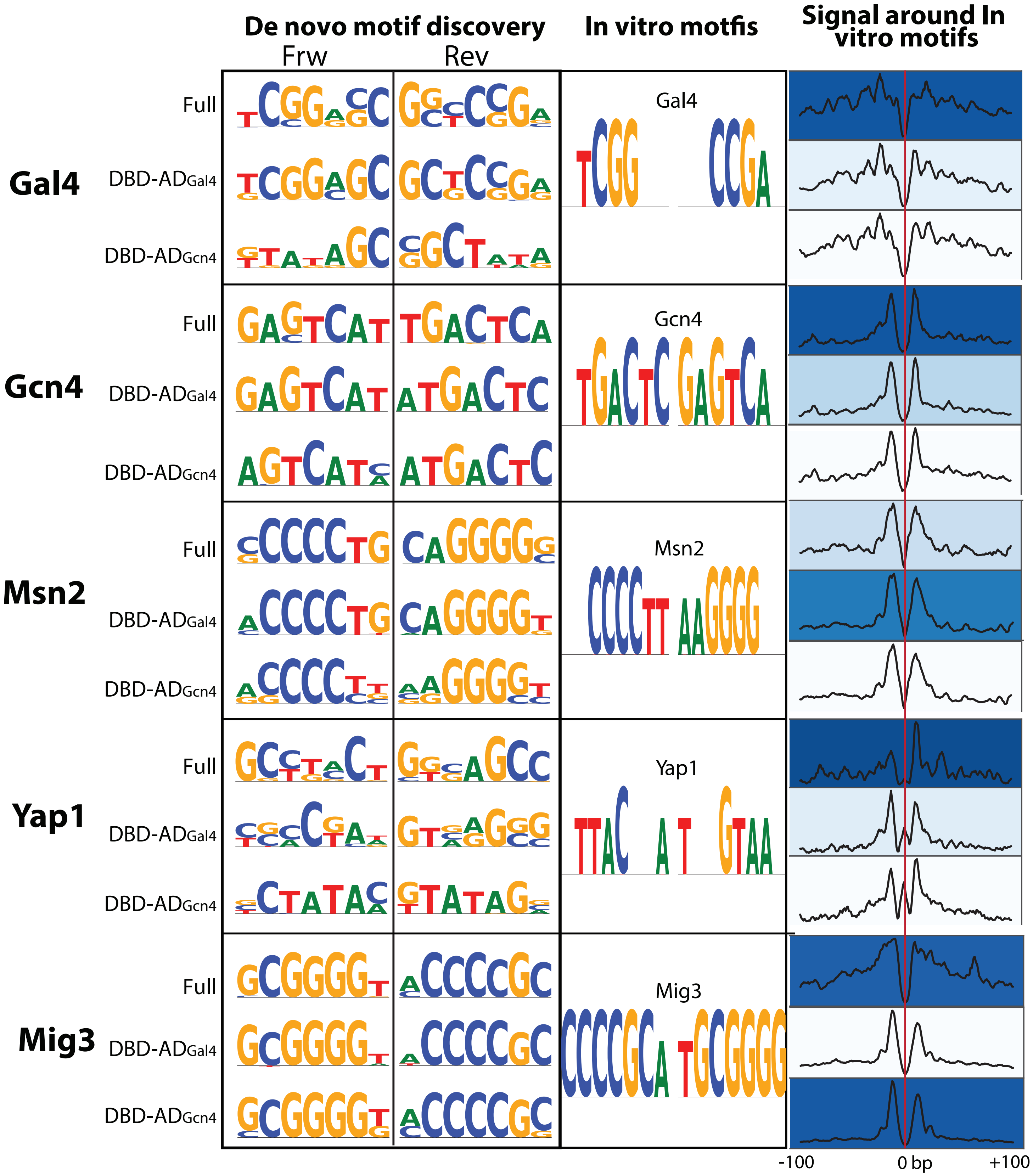

De novo motif discovery
Frw
Rev
In vitro motfis
Signal around In vitro motifs
Gal4
Gcn4
Msn2
Yap1
Mig3
Full
DBD-ADGal4
DBD-ADGcn4
-100
0 bp
+100

Figure S5. Minimal and full TFs localize to the previously defined motif of their DBD:

Shown on the left is the motif Seq-logos calculated based on the data collected in this study using ChEC-seq (methods). The previously measured DBD preferred motifs [2] are shown on the middle panel, and the signal received in our experiments around these same motifs located within promoter regions for each of the same DBD carrying TFs is shown on the right (methods).

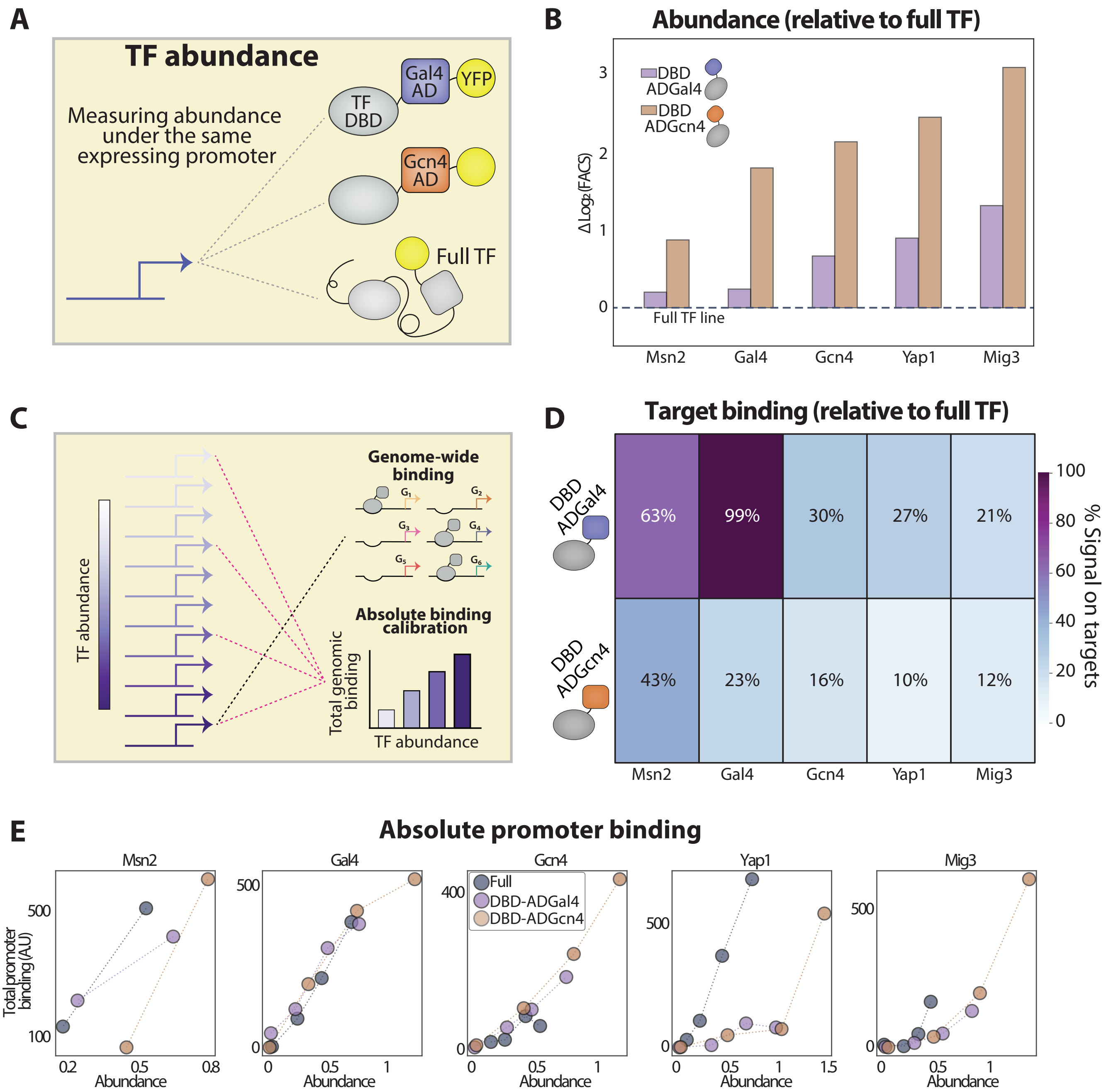

A
TF abundance
Measuring abundance under the same expressing promoter
Gal4 AD
YFP
TF DBD
Gcn4 AD
Full TF
B
Abundance (relative to full TF)
ΔLog2(FACS)
DBD ADGal4
DBD ADGcn4
Full TF line
Msn2
Gal4
Gcn4
Yap1
Mig3
C
TF abundance
Genome-wide binding
Absolute binding calibration
Total genomic binding
TF abundance
D
Target binding (relative to full TF)
DBD ADGal4
DBD ADGcn4
63%
99%
30%
27%
21%
43%
23%
16%
10%
12%
% Signal on targets
E
Absolute promoter binding
Total promoter binding (AU)
Abundance
Full
DBD-ADGal4
DBD-ADGcn4

Figure S6. DBD-AD fusions display reduced absolute binding at TF-bound promoters:

(A-B) *DBD-AD fusions reach higher protein abundance than full TFs while expressed under the same promoter.* For each selected TF, we generated a set of three strains expressing the respective DBD-ADs and full TFs under the same promoters. We compared their protein abundance using YFP-fusion and flow cytometry (A, scheme). The DBD-AD fusions reached a higher protein abundance in all five DBD-related sets as compared to the full TFs, as shown in (B), where the abundance of both minimal TFs is presented as the log2 fold-change from the abundance of the full TF. Note that factors carrying the AD_{Gcn4} were consistently more abundant than those carrying the AD_{Gal4}.

(C) *Measuring relative and absolute binding of the minimal AD-DBD TFs:* To measure relative binding to the native TF bound promoters, we mapped the binding profiles of the fifteen DBD-AD and full TFs above following their over-expression using the strong TEF1 promoter in strains deleted of the respective TF from the native locus. For measuring absolute promoter binding, we included 4 strains from each of our libraries (2 for DBD_{Msn2} containing strains) chosen to span the range of TF abundances and measured their binding while adding a calibration control (methods).

(D) *A lower fraction of DBD-AD binding signal localizes to native TF-bound promoters, as compared to full TFs:* Shown are the fractions of signal localized to the set of native TF-bound promoters, defined in Fig. 1B, that passed the induction score filter as described in Fig.2B. The values are presented as the signal received for the DBD-AD fusions relative to that of the full TF as measured without the calibration control. Note that the relative binding signal at TF-bound promoters is reduced in all DBD-AD fusions, excluding DBD_{Gal4}-AD_{Gal4}.

(E) *Absolute promoter binding may differ between minimal and full TFs:* Each indicated TF's calibrated total promoter binding is shown as a function of its measured protein abundance (presented as the $\log_{10}$ FC from non-fluorescent BY4741 cells, methods). Note the expected increase in promoter binding with increasing TF abundance. Also note that in the case of the Gcn4 and Gal4 DBDs, promoter binding appears similar, comparing the full to the minimal TFs expressed at the same levels. In the case of the other three DBDs tested, promoter binding by the full TF is significantly stronger than the binding of respective DBD-AD factors expressed under the same levels. Note that although the DBD-AD fusions are of higher abundance when expressed under the same promoter, their relative signal is shifted from the wild-type TF-defined targets, as shown in (D). Therefore, together with the observation that their calibrated absolute binding is equal to or lower than that of the respective full TF, we conclude that DBD-AD fusions display reduced absolute binding at promoters natively bound by the respective TF.

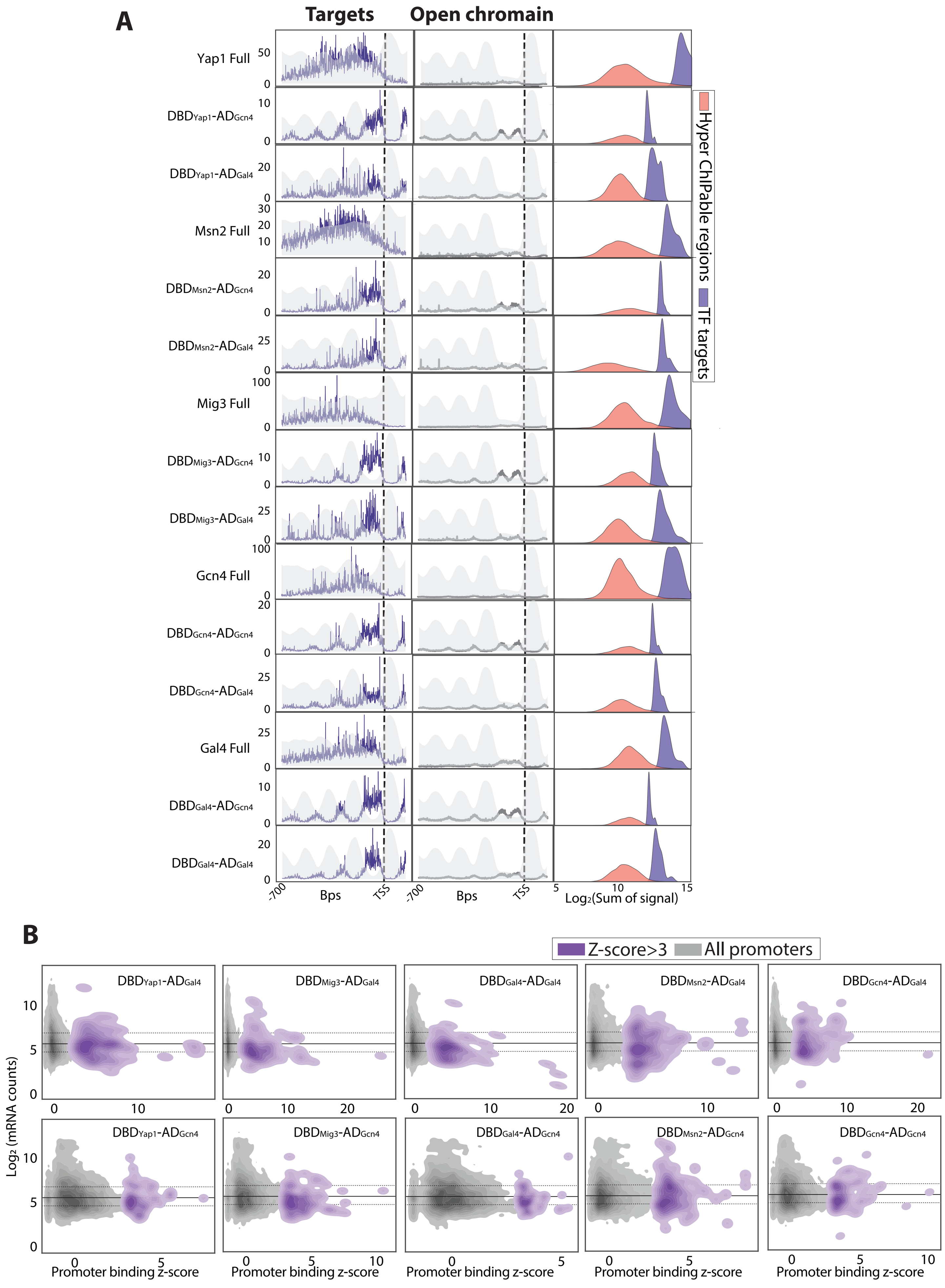

A
Targets
Open chromain
Yap1 Full
DBDYap1-ADGcn4
DBDYap1-ADGal4
Msn2 Full
DBDMsn2-ADGcn4
DBDMsn2-ADGal4
Mig3 Full
DBDMig3-ADGcn4
DBDMig3-ADGal4
Gcn4 Full
DBDGcn4-ADGcn4
DBDGcn4-ADGal4
Gal4 Full
DBDGal4-ADGcn4
DBDGal4-ADGal4
Hyper ChIPable regions
TF targets
-700
Bps
TSS
Log2(Sum of signal)
B
Z-score>3
All promoters
Log2 (mRNA counts)
Promoter binding z-score

Figure S7. The binding of minimal TFs to newly acquired promoters is not explained by open chromatin or high expression levels:

(A) *The signal of minimal TFs is not attributable to open chromatin:* Shown on the left is the average binding signal received for each of the indicated full TFs at their target promoters and of the indicated minimal TFs on their newly acquired ones, not bound by the respective full TF (methods). Shown in the middle for each TF is the average binding signal received on a same-sized group of promoters on which the nucleosome occupancy, defined by the sum of the signal received using MNase-seq [1], in 200 bps upstream to the TSS is the lowest amongst all promoters. Note the low signal received on these nucleosome-free promoters compared to the newly acquired targets of the DBD-AD minimal TFs. Shown on the right is the $\log_2$ sum of signals received on the same targets as in (A) compared to the binding signals received on hyper-CHiPable regions defined in [3]. Both types of regions are normalized to the same size.

(B) *The signal of minimal TFs cannot be explained by high expression of downstream genes:* Shown for each minimal TF indicated on top is the z-score of the sum of signal on promoters (x-axis) compared to the basal expression of the downstream gene (y-axis, methods). The distribution of target promoters of each minimal TF is purple (z-score>3), and the distribution of all other promoters is gray.

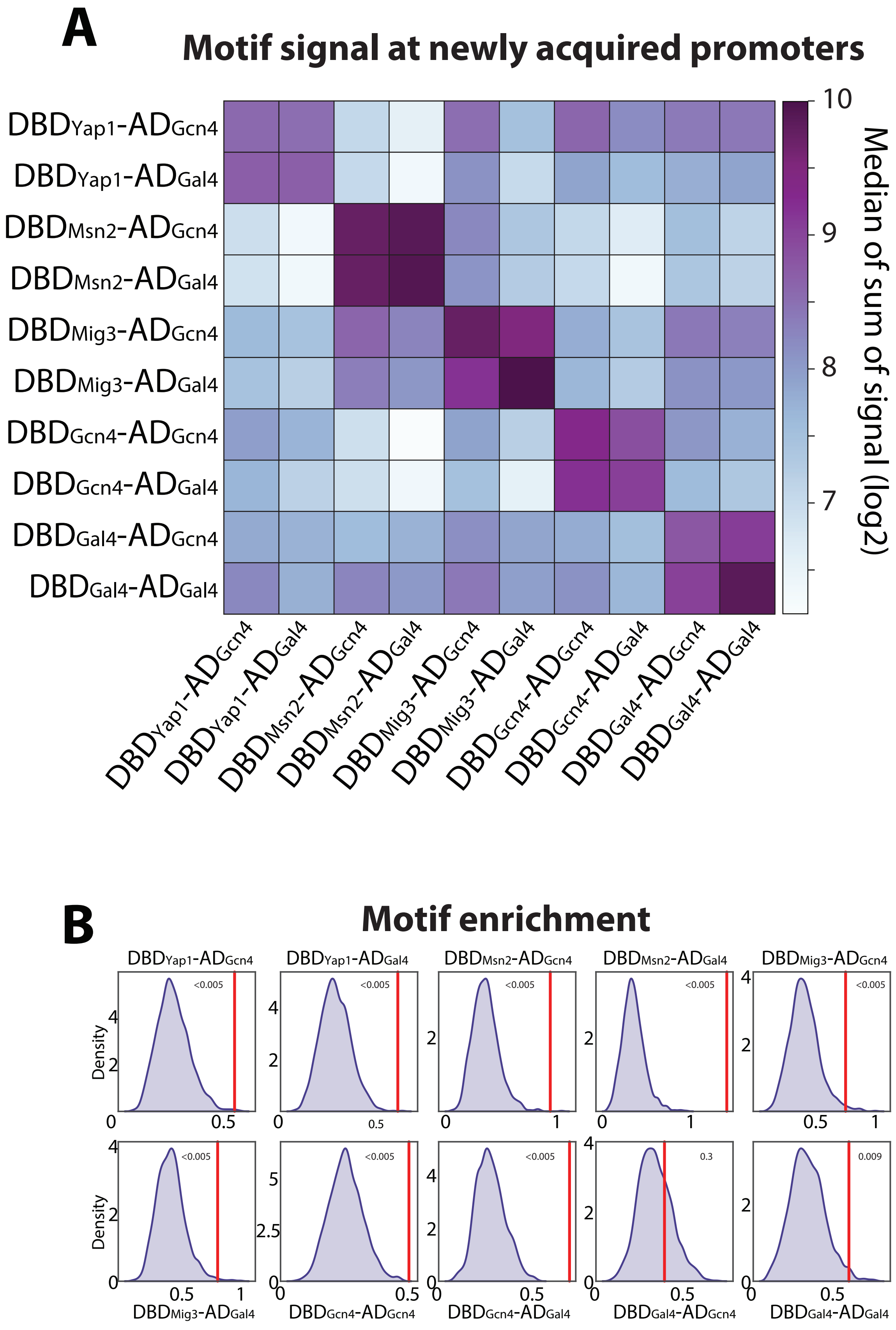
A
Motif signal at newly acquired promoters
DBDYap1-ADGcn4
DBDYap1-ADGal4
DBDMsn2-ADGcn4
DBDMsn2-ADGal4
DBDMig3-ADGcn4
DBDMig3-ADGal4
DBDGcn4-ADGcn4
DBDGcn4-ADGal4
DBDGal4-ADGcn4
DBDGal4-ADGal4
10
9
8
7
Median of sum of signal (log2)
B
Motif enrichment
Density
<0.005
0.3
0.009

Figure S8. The newly acquired DBD-AD target promoters are enriched with the motif of the corresponding DBD:

(A-B) Shown in (A) is the median sum of the signal received for each minimal TF indicated on the bottom on the preferred motif of the DBD (methods) of the minimal TF indicated on the left within its newly acquired promoters, not bound by the respective full TF (methods). For each minimal TF, the average number of DBD preferred motifs in its set of newly acquired promoters was calculated and is shown on (C, red vertical line). A similar-sized group of promoters was randomly sampled, and the average number of DBD-preferred motifs within this promoter group was calculated. This process was repeated 1000 times and is shown as a distribution. The z-test p-value is indicated on top.

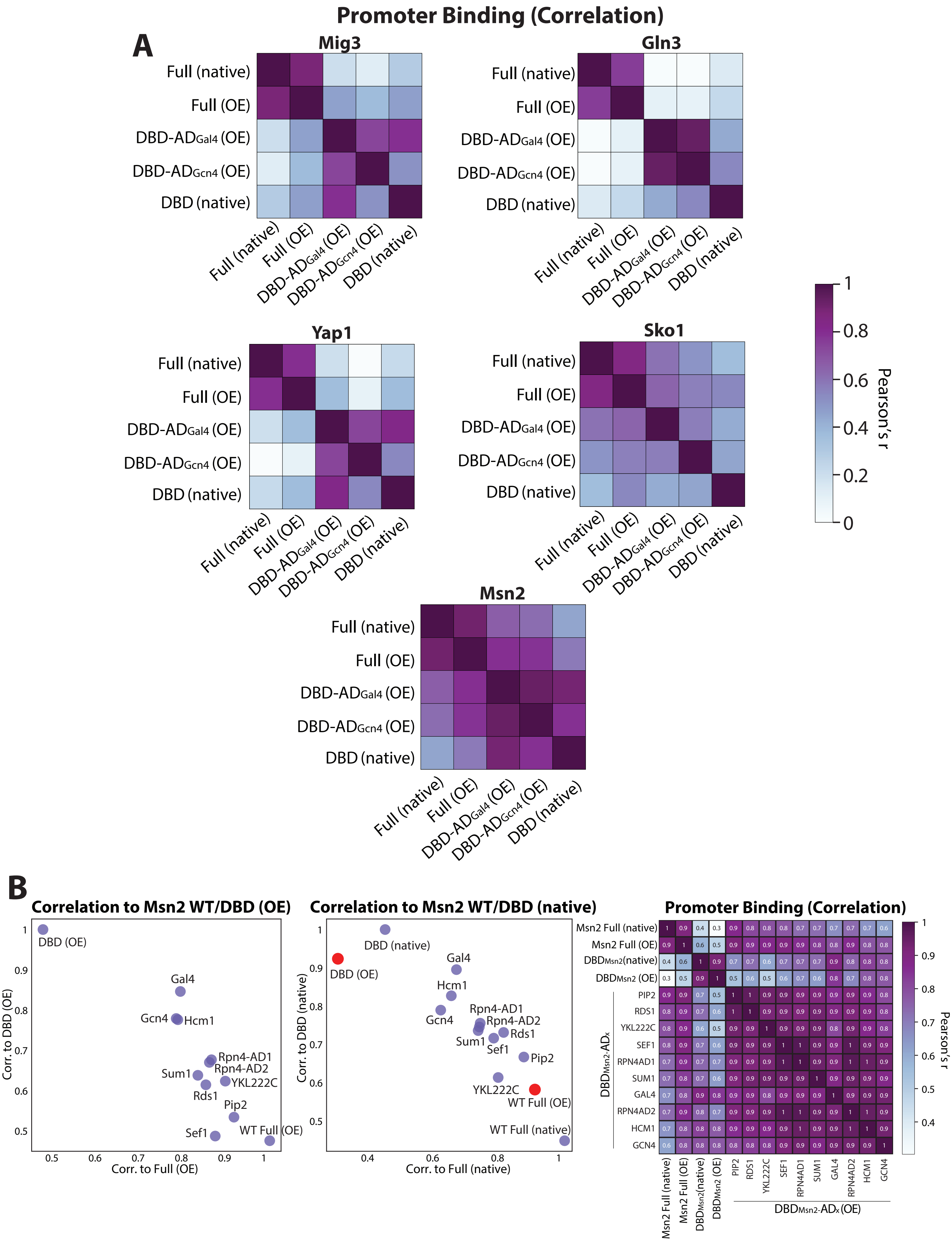

Promoter Binding (Correlation)
A
Mig3
Gln3
Yap1
Sko1
Msn2
Full (native)
Full (OE)
DBD-ADGal4 (OE)
DBD-ADGcn4 (OE)
DBD (native)
Pearson's r
B
Correlation to Msn2 WT/DBD (OE)
Corr. to DBD (OE)
Corr. to Full (OE)
DBD (OE)
Gal4
Gcn4
Hcm1
Rpn4-AD1
Rpn4-AD2
Sum1
YKL222C
Rds1
Pip2
Sef1
WT Full (OE)
Correlation to Msn2 WT/DBD (native)
Corr. to DBD (native)
Corr. to Full (native)
DBD (native)
WT Full (native)
Promoter Binding (Correlation)
Msn2 Full (native)
Msn2 Full (OE)
DBDMsn2(native)
DBDMsn2 (OE)
PIP2
RDS1
YKL222C
SEF1
RPN4AD1
SUM1
GAL4
RPN4AD2
HCM1
GCN4
DBDMsn2-ADx
DBDMsn2-ADx (OE)

Figure S9. Similarity of minimal TF binding profiles to the respective DBD and the influence of AD identity on DBD_{Msn2} binding:

(A) *DBD-AD fusions mostly resemble the binding profile of the respective DBD:* Shown is the correlation of the sum of signal over all promoters between the indicated factors. Note the high correlation between the DBDs of Mig3, Gln3, Yap1, and Msn2 (expressed under the native promoter) to the respective DBD-AD fusions. DBD Data from [4,5].

(B) *AD identity influences the genomic localization of DBD_{Msn2}-based minimal TFs*: Shown on the left is the correlation between DBD_{Msn2}-AD_x minimal TFs to the over-expressed full Msn2 (x-axis) and the over-expressed DBD_{Msn2} (y-axis). The identity of the AD of each fusion is also indicated. AD sequences are taken from [6] , and can be found in (Supplemental Table 1). Shown in the middle is the analysis comparing the DBD_{Msn2}-AD_x minimal TFs to the natively expressed Msn2 (x-axis) and the DBD-only mutant expressed under the native Msn2 promoter (y-axis)[5]. Red dots indicate the correlation between the over-expressed and natively expressed factors, as indicated in the text. Shown on the right is the correlation of the sum of the signal over all promoters for the indicated factors.

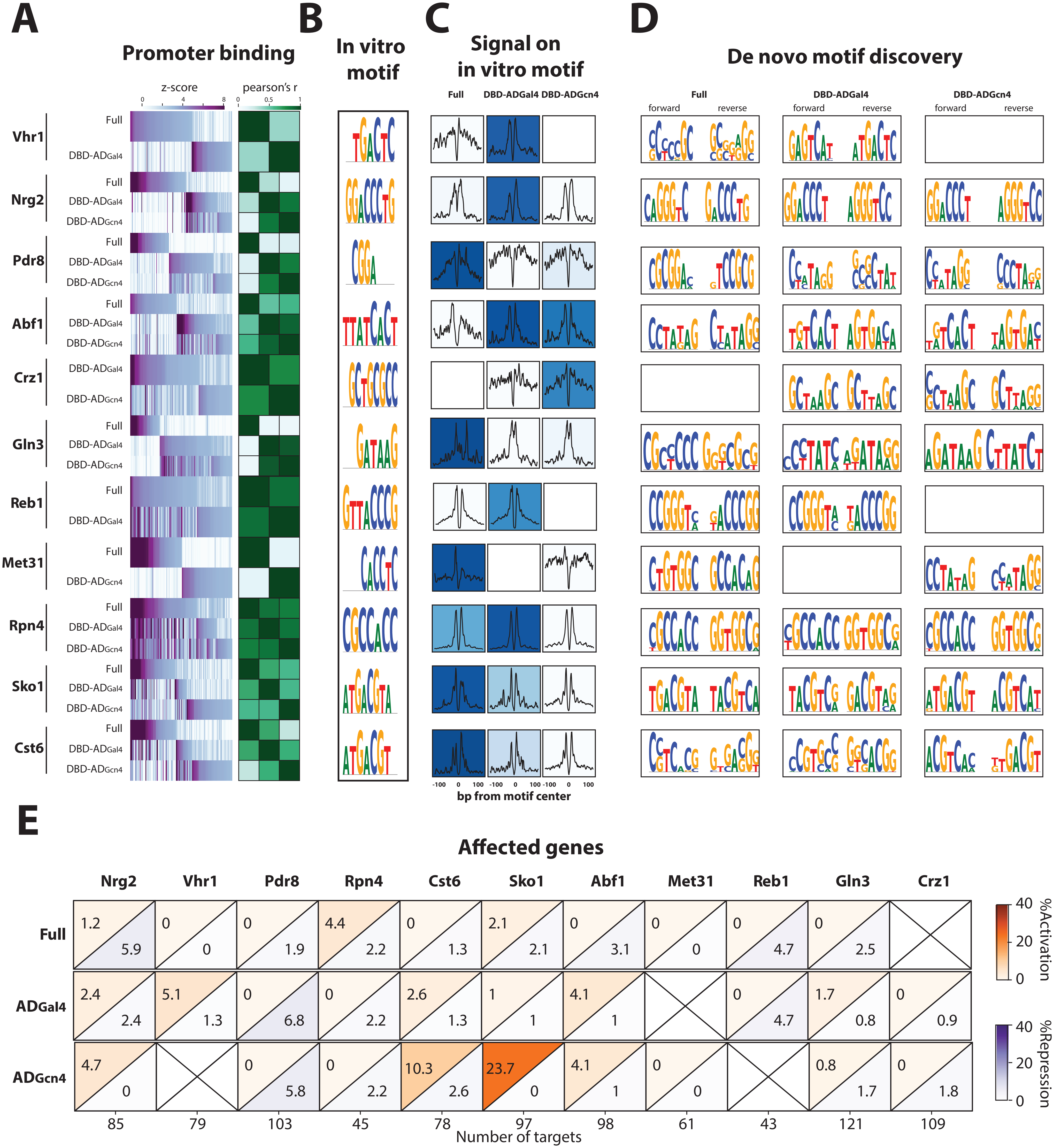
A
Promoter binding
z-score
0 4 8
pearson's r
0 0.5 1
Vhr1
Full
DBD-ADGal4
Nrg2
Full
DBD-ADGal4
DBD-ADGcn4
Pdr8
Full
DBD-ADGal4
DBD-ADGcn4
Abf1
Full
DBD-ADGal4
DBD-ADGcn4
Crz1
DBD-ADGal4
DBD-ADGcn4
Gln3
Full
DBD-ADGal4
DBD-ADGcn4
Reb1
Full
DBD-ADGal4
Met31
Full
DBD-ADGcn4
Rpn4
Full
DBD-ADGal4
DBD-ADGcn4
Sko1
Full
DBD-ADGal4
DBD-ADGcn4
Cst6
Full
DBD-ADGal4
DBD-ADGcn4
B
In vitro motif
C
Signal on in vitro motif
Full
DBD-ADGal4
DBD-ADGcn4
-100 0 100 -100 0 100 -100 0 100
bp from motif center
D
De novo motif discovery
Full
DBD-ADGal4
DBD-ADGcn4
forward
reverse
E
Affected genes
Nrg2 Vhr1 Pdr8 Rpn4 Cst6 Sko1 Abf1 Met31 Reb1 Gln3 Crz1
Full
1.2 5.9 | 0 0 | 0 1.9 | 4.4 2.2 | 0 1.3 | 2.1 2.1 | 0 3.1 | 0 0 | 0 4.7 | 0 2.5
ADGal4
2.4 2.4 | 5.1 1.3 | 0 6.8 | 0 2.2 | 2.6 1.3 | 1 1 | 4.1 1 | 0 4.7 | 1.7 0.8 | 0 0.9
ADGcn4
4.7 0 | 0 5.8 | 0 2.2 | 10.3 2.6 | 23.7 0 | 4.1 1 | 0 0 | 0.8 1.7 | 0 1.8
85 79 103 45 78 97 98 61 43 121 109
Number of targets
%Activation
0 20 40
%Repression
0 20 40

Figure S10. Prescreen minimal TFs also acquire new promoters and fail to induce their transcription:

(A) *Minimal TFs used in our prescreen localize to new promoters, not bound by the full TF:* Shown on the left, for each DBD-related TF set (rows), are all promoters (columns) bound by at least one indicated factor as measured by ChEC-seq. The correlation of their sum of the signal over all promoters in the genome is shown on the right. Note that the binding of the indicated factors is measured in cells expressing the endogenous TF, except for the DBD_{Rpn4} and DBD_{Gln3} related sets of strains in which the endogenous TFs were deleted (methods, Supplemental Table 1).

(B-D) *Despite localizing to new promoters, motif selection is similar:* Shown in (B) is the corrected *in vitro* preferred DBD motif of each TF (methods), and in (C), the signal received in our study around these same motifs found within promoter regions for each of the same-DBD carrying TFs. Motif Seq-logos, calculated based on our data, are shown in (D, methods).

(E) *DBD-AD fusions rarely induce expression of newly bound genes:* Shown for all tested full and minimal TFs are the fraction of induced (upper triangle, brown) and repressed genes (lower triangle, blue) on the joint set of newly bound promoters of each of the same DBD-related minimal TFs. Note the distinctly high induction rate of the DBD_{sko1} and DBD_{Cst6} fusion to the AD_{Gcn4}. A black "X" marks missing strains.

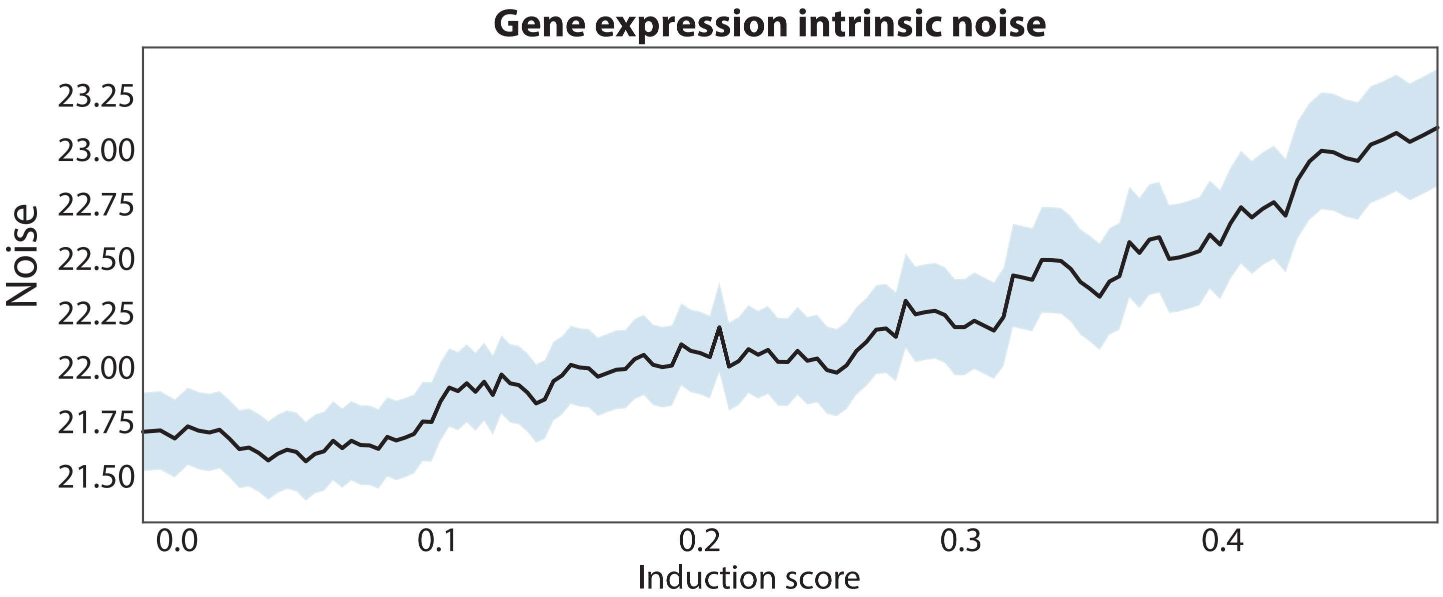

Gene expression intrinsic noise
Noise
23.25
23.00
22.75
22.50
22.25
22.00
21.75
21.50
0.0
0.1
0.2
0.3
0.4
Induction score

Figure S11. Gene induction scores correlate with expression noise:

The average expression noise is shown as a function of the sorted averaged induction score measured in our promoter library experiments (presentation as in Fig. 4 C-G, methods).

A

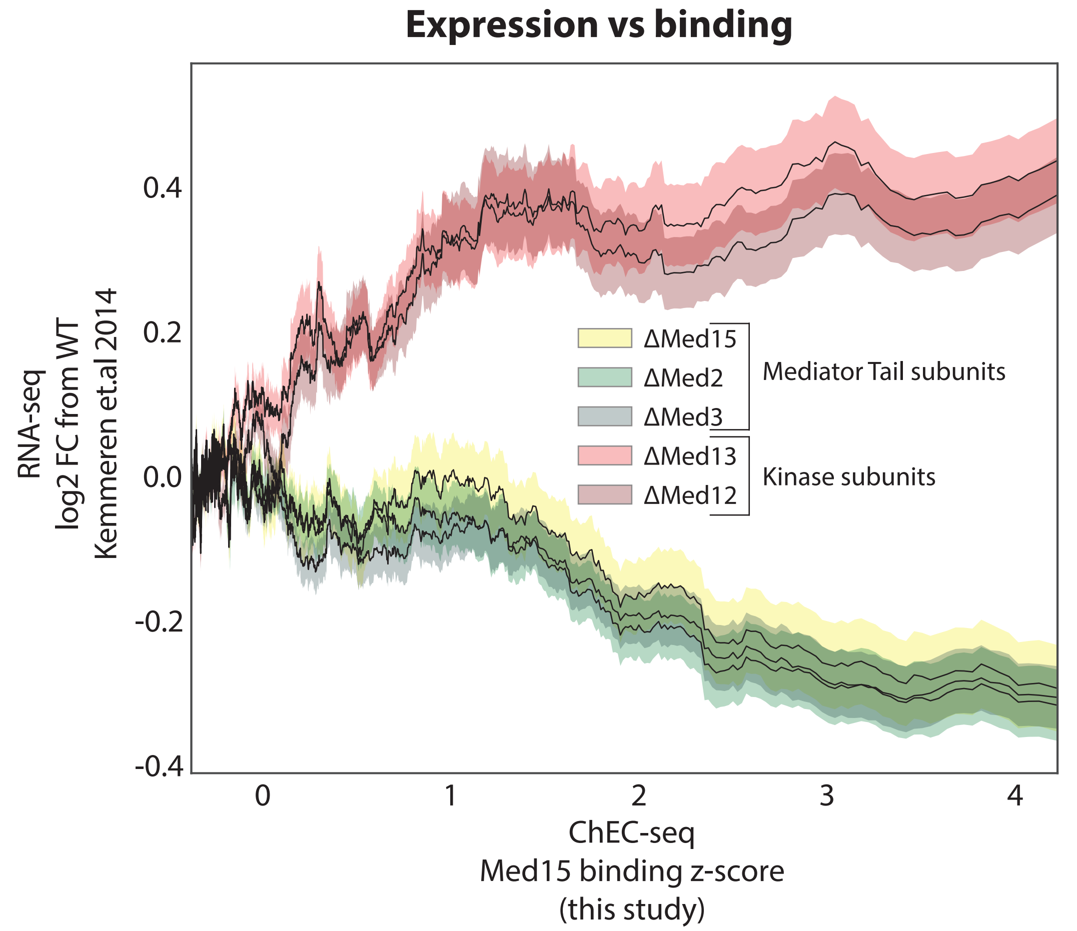
Expression vs binding
RNA-seq
log2 FC from WT
Kemmeren et.al 2014
0.4
0.2
0.0
-0.2
-0.4
ΔMed15
ΔMed2
ΔMed3
ΔMed13
ΔMed12
Mediator Tail subunits
Kinase subunits
0
1
2
3
4
ChEC-seq
Med15 binding z-score
(this study)

B

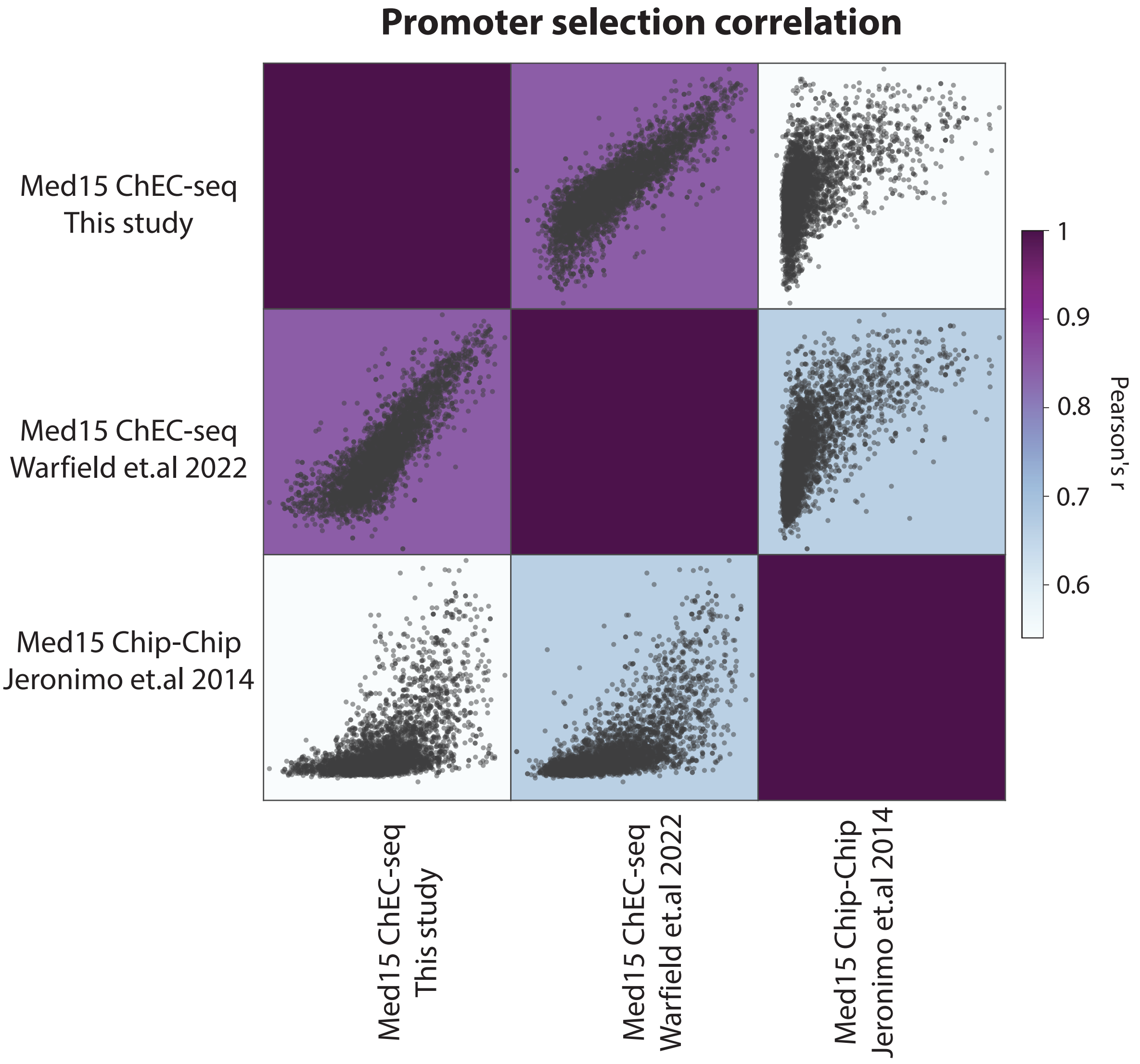
Promoter selection correlation
Med15 ChEC-seq
This study
Med15 ChEC-seq
Warfield et.al 2022
Med15 Chip-Chip
Jeronimo et.al 2014
1
0.9
0.8
0.7
0.6
Pearson's r
Med15 ChEC-seq
This study
Med15 ChEC-seq
Warfield et.al 2022
Med15 Chip-Chip
Jeronimo et.al 2014

Figure S12. Med15 localization corresponds with previously published datasets:

(A) *Med15 binding profiles correlate with gene expression data collected in a previous study:* Shown is the average effect of a deletion of the indicated Mediator subunits on gene expression as measured in [7] as a function of Med15 binding collected in our study (methods). Note the decrease in expression of highly bound Med15 targets when deleting Mediator tail subunits and the increase in expression when deleting the subunits of the Mediator kinase module in charge of inhibiting gene induction by Mediator.

(B) *Med15 binding profiles resemble those collected by other studies:* Shown is the correlation of the sum of signal over all promoters between Med15 binding data generated in this study to profiles generated on two previously published ones. Data from [8] was collected using ChEC-seq, and data from [9] was collected using ChIP-chip.

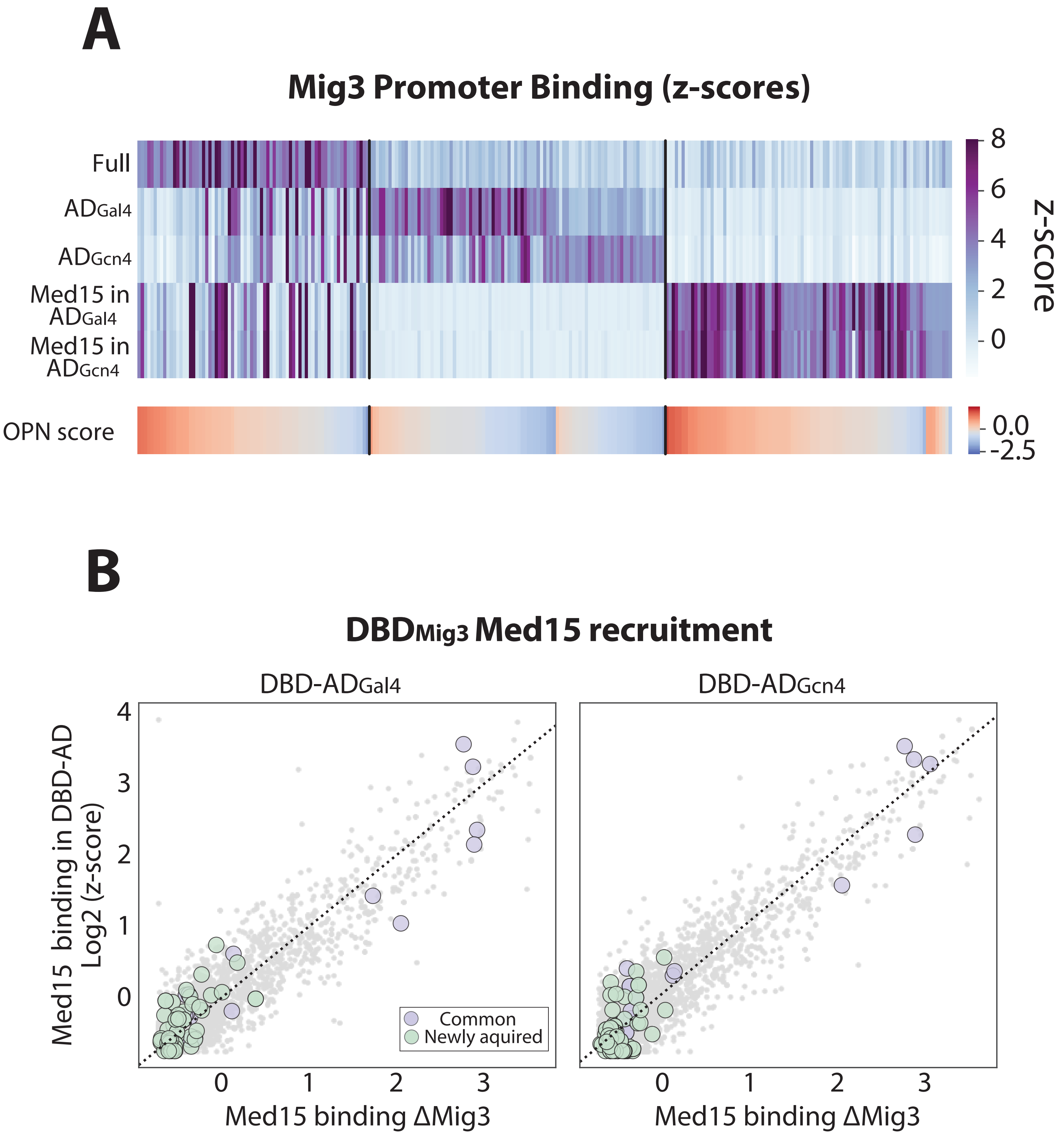
A
Mig3 Promoter Binding (z-scores)
Full
ADGal4
ADGcn4
Med15 in ADGal4
Med15 in ADGcn4
OPN score
z-score
8
6
4
2
0
0.0
-2.5
B
DBDMig3 Med15 recruitment
DBD-ADGal4
DBD-ADGcn4
Med15 binding in DBD-AD Log2 (z-score)
Med15 binding ΔMig3
Common
Newly aquired

Figure S13. DBD_{Mig3}-AD fusions fail to recruit Med15 to newly acquired promoters:

(A-B) Med15 binding was measured in the indicated strains using ChEC-seq. Shown are all promoters (rows) bound in at least one DBD-related TF (rows, z-score > 3) as in Fig. 5C. Note the inability of the DBD-AD fusions to recruit Med15 to their newly acquired bound promoters. Shown in (B) is the recruitment of Med15 to common and newly acquired binding targets of the DBD_{Mig3}-AD in the indicated strain (y-axis) as compared to a strain lacking this same minimal TF and deleted of the native Mig3 (x-axis) as in Fig. 5D.

A

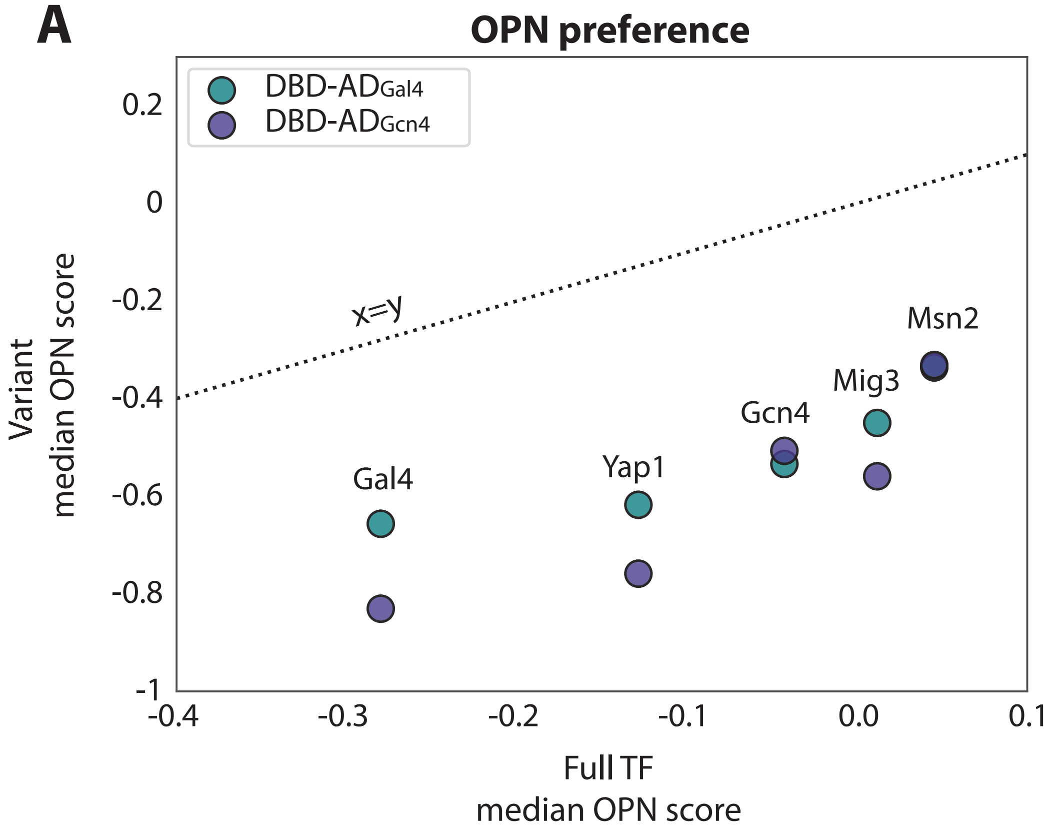
OPN preference
DBD-ADGal4
DBD-ADGcn4
x=y
Msn2
Mig3
Gcn4
Yap1
Gal4
0.2
0
-0.2
-0.4
-0.6
-0.8
-1
-0.4
-0.3
-0.2
-0.1
0.0
0.1
Variant
median OPN score
Full TF
median OPN score

B

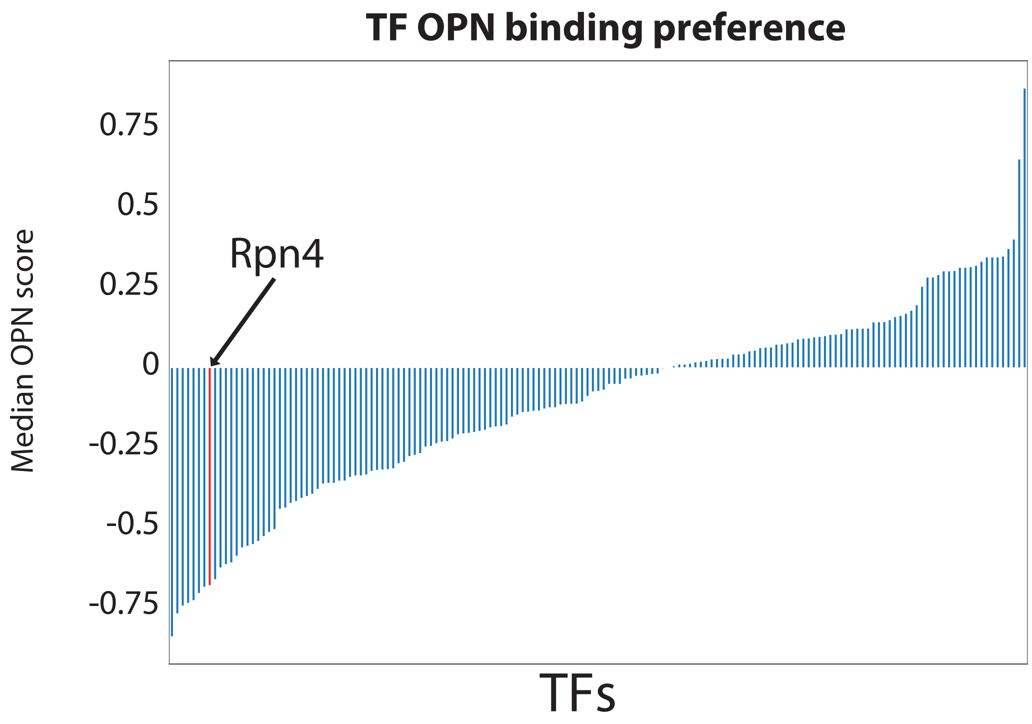
TF OPN binding preference
Rpn4
0.75
0.5
0.25
0
-0.25
-0.5
-0.75
Median OPN score
TFs

Figure S14. DBD-AD fusions prefer binding low OPN-score promoters, as compared to the full TFs:

(A) *Minimal TFs show a preference for binding promoters of low OPN-score:* Shown is the median OPN score of bound promoters (z-score > 3) of each DBD-AD compared to the average OPN score of the bound targets of the respective DBD-containing full TF. The dashed line represents equal OPN preferences of the Full and minimal TFs. Note that the targets of all minimal TFs are of lower OPN score.

(B) *The native Rpn4 TF binds low OPN-score targets:* Shown is the average OPN score of the targets of each of the 145 yeast TFs of our lab dataset. The location of Rpn4 is marked by an arrow, showing its preferential binding to low-OPN score promoters.

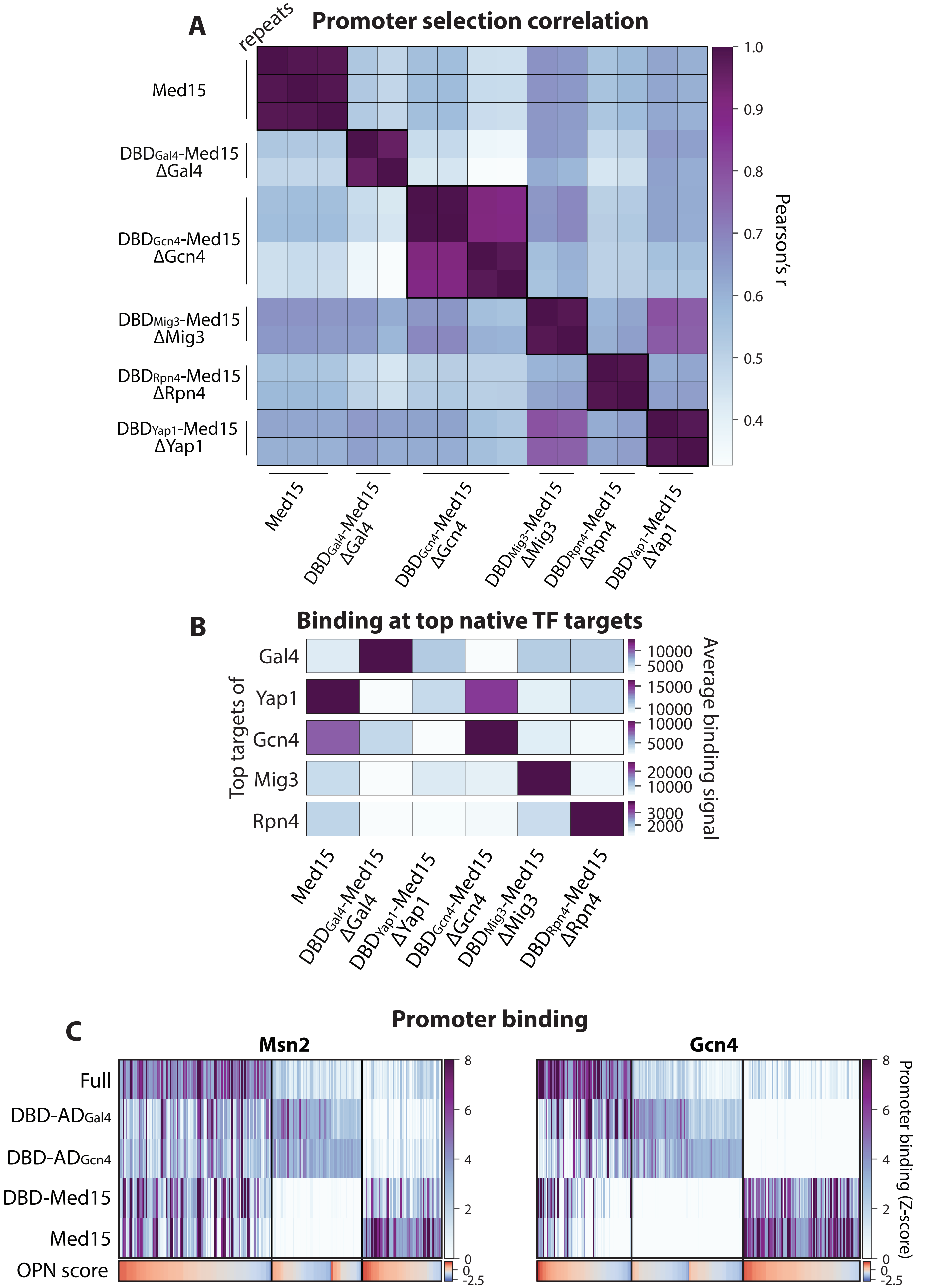

A
Promoter selection correlation
repeats
Med15
DBDGal4-Med15 ΔGal4
DBDGcn4-Med15 ΔGcn4
DBDMig3-Med15 ΔMig3
DBDRpn4-Med15 ΔRpn4
DBDYap1-Med15 ΔYap1
Pearson's r
1.0
0.9
0.8
0.7
0.6
0.5
0.4
B
Binding at top native TF targets
Top targets of
Gal4
Yap1
Gcn4
Mig3
Rpn4
Average binding signal
10000
5000
15000
10000
10000
5000
20000
10000
3000
2000
Med15
DBDGal4-Med15 ΔGal4
DBDYap1-Med15 ΔYap1
DBDGcn4-Med15 ΔGcn4
DBDMig3-Med15 ΔMig3
DBDRpn4-Med15 ΔRpn4
C
Promoter binding
Msn2
Gcn4
Full
DBD-ADGal4
DBD-ADGcn4
DBD-Med15
Med15
OPN score
Promoter binding (Z-score)
8
6
4
2
0
-2.5

Figure S15. DBD identity influences DBD-Med15 genomic localization:

(A-B) *The identity of the DBD affects the binding profiles of Med15-based fusions:* Shown in (A) is the correlation of the sum of signal over all promoters for the indicated factors. Note the high correlation within technical repeats compared to the mild correlation between fusions of Med15 to different DBDs. For each wild-type TF, the 10 top-bound targets were selected. Shown in (B) is the mean signal received for the Med15-DBD fusion indicated on the bottom, on the set of targets of the TF indicated on the left. The average signal of the native Med15 over these promoter sets is also shown.

(C) *DBD-Med15 fusions do not localize to the newly acquired DBD-AD promoters also when natively expressed:* We fused the endogenous Med15 to the DBDs of Msn2 and Gcn4. The binding at target promoters of the indicated full and minimal factors is shown as in Fig. 5C. The red line separates the binding targets of the full TFs from the newly acquired DBD-AD targets.

Table S1. Summary of strains analyzed in this study

Presented are the complete information on the creation and genotypes of the yeast strains used in this study, the assays performed on them, essential DNA sequences, and the list of primers used to create them.

[1] R. Bar-Ziv, S. Brodsky, M. Chapal, N. Barkai, Transcription Factor Binding to Replicated DNA, Cell Reports. 30 (2020) 3989-3995.e4. https://doi.org/10.1016/j.celrep.2020.02.114.

[2] M.T. Weirauch, A. Yang, M. Albu, A.G. Cote, A. Montenegro-Montero, P. Drewe, H.S. Najafabadi, S.A. Lambert, I. Mann, K. Cook, H. Zheng, A. Goity, H. van Bakel, J.-C. Lozano, M. Galli, M.G. Lewsey, E. Huang, T. Mukherjee, X. Chen, J.S. Reece-Hoyes, S. Govindarajan, G. Shaulsky, A.J.M. Walhout, F.-Y. Bouget, G. Ratsch, L.F. Larrondo, J.R. Ecker, T.R. Hughes, Determination and inference of eukaryotic transcription factor sequence specificity, Cell. 158 (2014) 1431–1443. https://doi.org/10.1016/j.cell.2014.08.009.

[3] L. Teytelman, D.M. Thurtle, J. Rine, A. van Oudenaarden, Highly expressed loci are vulnerable to misleading ChIP localization of multiple unrelated proteins, Proc Natl Acad Sci U S A. 110 (2013) 18602–18607. https://doi.org/10.1073/pnas.1316064110.

[4] D.K. Kumar, F. Jonas, T. Jana, S. Brodsky, M. Carmi, N. Barkai, Complementary strategies for directing in vivo transcription factor binding through DNA binding domains and intrinsically disordered regions, Mol Cell. 83 (2023) 1462-1473.e5. https://doi.org/10.1016/j.molcel.2023.04.002.

[5] S. Brodsky, T. Jana, K. Mittelman, M. Chapal, D.K. Kumar, M. Carmi, N. Barkai, Intrinsically Disordered Regions Direct Transcription Factor In Vivo Binding Specificity, Molecular Cell. 79 (2020) 459-471 4. https://doi.org/10.1016/j.molcel.2020.05.032.

[6] A.L. Sanborn, B.T. Yeh, J.T. Feigerle, C.V. Hao, R.J. Townshend, E. Lieberman Aiden, R.O. Dror, R.D. Kornberg, Simple biochemical features underlie transcriptional activation domain diversity and dynamic, fuzzy binding to Mediator, eLife. 10 (2021) e68068. https://doi.org/10.7554/eLife.68068.

[7] P. Kemmeren, K. Sameith, L.A.L. van de Pasch, J.J. Benschop, T.L. Lenstra, T. Margaritis, E. O'Duibhir, E. Apweiler, S. van Wageningen, C.W. Ko, S. van Heesch, M.M. Kashani, G. Ampatziadis-Michailidis, M.O. Brok, N.A.C.H. Brabers, A.J. Miles, D. Bouwmeester, S.R. van Hooff, H. van Bakel, E. Sluiters, L.V. Bakker, B. Snel, P. Lijnzaad, D. van Leenen, M.J.A. Groot Koerkamp, F.C.P. Holstege, Large-Scale Genetic Perturbations Reveal Regulatory Networks and an Abundance of Gene-Specific Repressors, Cell. 157 (2014) 740–752. https://doi.org/10.1016/j.cell.2014.02.054.

[8] L. Warfield, R. Donczew, L. Mahendrawada, S. Hahn, Yeast Mediator facilitates transcription initiation at most promoters via a Tail-independent mechanism, Mol Cell. 82 (2022) 4033-4048.e7. https://doi.org/10.1016/j.molcel.2022.09.016.

[9] C. Jeronimo, F. Robert, Kin28 regulates the transient association of Mediator with core promoters, Nat Struct Mol Biol. 21 (2014) 449–455. https://doi.org/10.1038/nsmb.2810.
